# Research progress of ginseng in the treatment of gastrointestinal cancers

**DOI:** 10.3389/fphar.2022.1036498

**Published:** 2022-10-12

**Authors:** Baoyi Ni, Xiaotong Song, Bolun Shi, Jia Wang, Qianhui Sun, Xinmiao Wang, Manman Xu, Luchang Cao, Guanghui Zhu, Jie Li

**Affiliations:** ^1^ Guang’anmen Hospital, China Academy of Chinese Medical Sciences, Beijing, China; ^2^ Hongqi Hospital of Mudanjiang Medical University, Mudanjiang, China

**Keywords:** ginseng, ginsenosides, gastrointestinal tumours, molecular mechanism, natural medicine

## Abstract

Cancer has become one of the major causes of human death. Several anticancer drugs are available; howeve their use and efficacy are limited by the toxic side effects and drug resistance caused by their continuous application. Many natural products have antitumor effects with low toxicity and fewer adverse effects. Moreover, they play an important role in enhancing the cytotoxicity of chemotherapeutic agents, reducing toxic side effects, and reversing chemoresistance. Consequently, natural drugs are being applied as potential therapeutic options in the field of antitumor treatment. As natural medicinal plants, some components of ginseng have been shown to have excellent efficacy and a good safety profile for cancer treatment. The pharmacological activities and possible mechanisms of action of ginseng have been identified. Its broad range of pharmacological activities includes antitumor, antibacterial, anti-inflammatory, antioxidant, anti-stress, anti-fibrotic, central nervous system modulating, cardioprotective, and immune-enhancing effects. Numerous studies have also shown that throuth multiple pathways, ginseng and its active ingredients exert antitumor effects on gastrointestinal (GI) tract tumors, such as esophageal, gastric, colorectal, liver, and pancreatic cancers. Herein, we introduced the main components of ginseng, including ginsenosides, polysaccharides, and sterols, etc., and reviewed the mechanism of action and research progress of ginseng in the treatment of various GI tumors. Futhermore, the pathways of action of the main components of ginseng are discussed in depth to promote the clinical development and application of ginseng in the field of anti-GI tumors.

## 1 Introduction

Recently, the incidence of digestive tract tumors has increased rapidly as people’s lifestyles have changed dramatically and stress and poor eating habits have overwhelmed the digestive tract. Gastrointestinal (GI) tumors are a major disease threatening human life and health. According to the latest global cancer burden data for 2020 released by the International Agency for Research on Cancer of the World Health Organization, among the top ten tumors in terms of incidence, four are GI tumors, namely colorectal cancer (1.93 million, 10%), stomach cancer (1.09 million, 5.6%), liver cancer (0.91 million, 4.7%), and esophageal cancer (0.6 million, 3.1%); among the top ten tumors in terms of mortality Among the top 10 tumors, 5 are GI tract tumors, namely colorectal cancer (940,000, 9.4%), liver cancer (830,000, 8.3%), stomach cancer (770,000, 7.7%), esophageal cancer (540,000, 5.5%), and pancreatic cancer (470,000, 4.7%). In addition, GI tract tumors include cardia cancer, gastric mesenchymal tumor, mucinous adenocarcinoma of the appendix, duodenal cancer, gallbladder cancer, bile duct cancer, and anal cancer. Currently, surgery-centered regimens combined with chemotherapy are the cornerstone of multiple tumor treatment modalities. Chemotherapy, as one of the main treatments for GI tumors, usually produces significant therapeutic effects but is accompanied by non-negligible toxic effects. As the number of chemotherapy cycles continues to increase, tumor cells become less sensitive to chemotherapeutic agents and develop chemoresistance, leading to tumor recurrence and metastasis, directly affecting the near-term efficiency and long-term survival of patients (Liu Y et al., 2021).

An increasing number of natural products have antitumor effects with low toxicity and fewer adverse effects and are being applied as potential therapeutic options in the field of antitumor therapy. Moreover, numerous basic and clinical studies have shown that natural products play an important role in enhancing the cytotoxicity of chemotherapeutic agents, reducing toxic side effects, and resistance to chemotherapy. For example, some components of ginseng have been shown to have excellent efficacy and a good safety profile in cancer treatment (de Oliveira et al., 2018).

*Panax ginseng* C. A. Mey. is a herb belonging to the genus Ginseng, commonly referred to as “Ren shen” at Chinese. The genus Ginseng originated in the ancient tropics of the Tertiary Period, and there are five main species in the genus Ginseng: *Panax ginseng*, *Panax pseudoginseng*, *Panax japonicus*, *Rhizoma Panacis Majoris*, and *Panax quinquefolium*. Over the years, these plants have been widely studied and used in food or medicinal herbs, especially in many Asian countries with a much longer history, the main sources of which are China, the Korean Peninsula, Japan and Eastern Russia. Ginseng has a wide range of uses and, to date, remains popular. The pharmacological activities and possible mechanisms of action of ginseng and its active ingredients have been discovered in the past decades. Its broad range of pharmacological activities includes antibacterial, anti-inflammatory, antioxidant, anti-stress, anti-fibrotic, central nervous system modulation, cardioprotective, immune-enhancing effects, and significant antitumor activity (Shi et al., 2019). In addition, ginseng and its active ingredients have been shown to exert antitumor effects on various GI tumors, such as esophageal, gastric, colorectal, liver, and pancreatic cancers, through multiple pathways. In this review, the ethnopharmacology and main active ingredients of ginseng are presented, along with the antitumor evaluation of individual ginseng components, to reveal their respective modes of action in the field of GI tumors for prevention and treatment.

### 1.1 Main active ingredients responsible for the anti-tumor effect of ginseng

**FIGURE 1:**
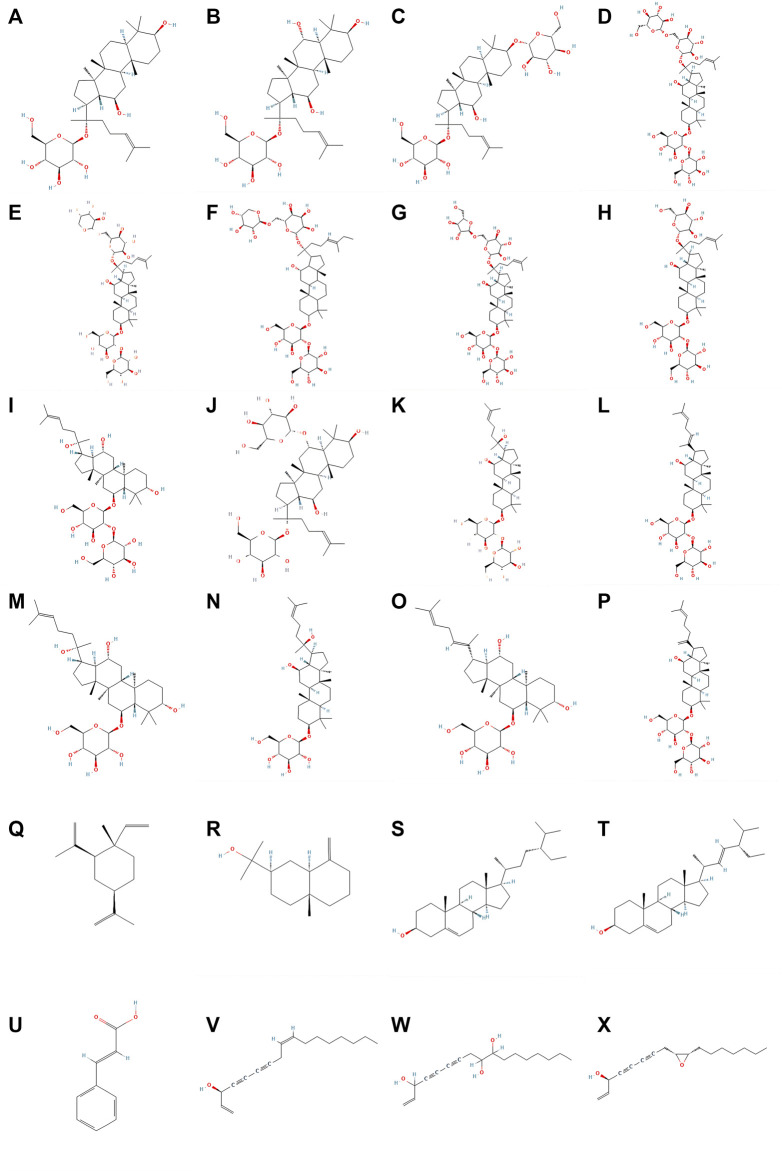
Molecular structureal formula of the main active ingredients of ginseng. **(A)** Ginsenosides-C-K **(B)** Ginsenosides-F1 **(C)** Ginsenosides-F2 **(D)** Ginsenosides-Rb1 **(E)** Ginsenosides-Rb2 **(F)** Ginsenosides-Rb3 **(G)** Ginsenosides-Rc **(H)** Ginsenosides-Rd **(I)** Ginsenosides-Rf **(J)** Ginsenosides-Rg1 **(K)** Ginsenosides-Rg3 **(L)** Ginsenosides-Rg5 **(M)** Ginsenosides-Rh1 **(N)** Ginsenosides-Rh2 **(O)** Ginsenosides-Rh4 **(P)** Ginsenosides-Rk1 **(Q)** beta-Elemene **(R)** beta-Eudesmol **(S)** beta-Sitosterol **(T)** Stigmasterol **(U)** Cinnamic_acid **(V)** Panaxynol **(W)** Panaxytriol **(X)** Panaxydol (Image credit: National Library of Medicine - National Center for Biotechnology Information https://pubchem.ncbi.nlm.nih.gov/).

The bioactive components of ginseng mainly include ginsenosides, ginseng polysaccharides (GPS), ginseng polyacetylenes, sterols, volatile oils, proteins, ginseng polypeptides, amino acids, vitamins, organic acids, and trace elements. The main antitumor components include ginsenosides, ginseng polysaccharides, ginseng polyacetylenes, sterols, and volatile oils (Hong et al., 2021). Among these, ginsenosides are the most important components of ginseng that exert antitumor effects. The molecular structural formulae of the main components of ginseng are shown in Figure 1.

#### 1.1.1 Ginsenosides

Ginsenosides are the main physiological activities and active ingredients of ginseng. They are triterpenoids and are classsified into three types according to their saponin skeleton and structure: dammaranes, oleanolic acids and oxytetracyclines. Among the three types of saponins, dammarane saponins are the most important. Dammarane saponins are further classified into protopanaxadiol (PPD) and protopanaxatriol (PPT). The saponin components are: 20(s)-PPD and 20(S)-PPT. Ginseng contains 45–60% PPD, 12–20% PPT, and 7–10% oleanolic saponins. Among them, ginsenosides Rb1, Rb2, Rb3, Rc, Rd, Rg3, Rg5, Rh2, Rs11, Rk1, F2, and C-K belong to panaxadiol saponins (PDS), whereas ginsenosides Re, Re7, Rg1, 6-acetyl-Rg3, Rg18, Rh1, Rh4, Rp1, Rf and F1,etc. are ginsenosides of the triol type (panaxadiol saponin, PTS) (Hong et al., 2021). Recently, some studies have proposed that ginsenosides can inhibit cell proliferation, induce apoptosis, inhibit Wnt/β-catenin, Nuclear factor-κB (NF-κB), EGFR/AKT, Janus kinase 2/signal transducer and activator of transcription 3 (JAK2/STAT3), PI3K/Akt/mTOR and MAPK/ERK. Moreover activation of these signaling pathway has a strong anti-tumor effect on the growth of various tumors, and can be used synergistically with various chemotherapy drugs to improve efficacy and reduce toxicity significantly. Thus, ginsenosides have broad development prospects as novel anticancer therapeutic agents and anticancer adjuvant drugs.

#### 1.1.2 GPS

Polysaccharides are a class of biological macromolecules with complex structures. Immunological activity is an important biological activity of polysaccharides, and many of which have been widely used clinically as immunity-enhancing drugs. GPS is a polymeric acidic polysaccharide extracted from ginseng. According to traditional Chinese medicine theory, GPS nourishes the “qi” of the spleen and lungs. The most effective pharmacological effect of GPS is immunity enhancement and improvement of the pathological state of the entire organism (Zhang E et al., 2017).

GPS, with a content of approximately 5%, is an important bioactive component of ginseng. It is a polymeric glucan composed of two parts: ginseng neutral sugar and ginseng acidic pectin, with ginseng starch accounting for approximately 80% of the total polysaccharide and ginseng pectin for approximately 20%. Neutral sugars mainly comprise amylose glucan and a small amount of arabinogalactan (AG). Acidic pectin is often a heteropolysaccharide rich in galacturonic acid. Ginseng polysaccharide, an indispensable chemical component of ginseng, is an early studied polysaccharide bioactive component, a light yellow to yellowish brown powder soluble in hot water. Various antitumor polysaccharides have been isolated and purified from the roots or fruits of ginseng plants, including GPS GFP1, PGP2a, PGPW1, ginseng, WGPA-1-HG, 2-HG, 3-HG, 4-HG, WGPA-3-RG, and 4-RG.

Ginseng pectin is the main pharmacologically active substance of GPS, which has various pharmacological effects, including antitumor, immunomodulatory, hypoglycemic, anti-radiation, anti-adhesive, anti-viral, antioxidant, inflammatory lowering and anti-septic effects. It is commonly used clinically for the comprehensive treatment of many types of malignant tumors, and to alleviate the adverse effects caused by chemotherapy and radiotherapy.

#### 1.1.3 Polyacetylenes

Polyacetylene (PA) is a lipophilic phytoconstituent with a wide range of biological activities. Medicinal plants containing alkynols have a long been used in Chinese medicine and are widely used in Asia (Cai et al., 2007). Panaxydol (PND), panaxytriol (PNT), and panaxynol (PNN) are the main polyacetylenes in ginseng that exhibit anticancer activity.

#### 1.1.4 Sterols

The main sterols components in ginseng are β-sitosterol and stigmasterol. Sterols reportedly inhibit the development and progression of many cancers, such as breast, prostate, colon, lung, stomach, and ovarian cancers, through various cell signaling pathways (Bin and Ameen, 2015; Mahmoud and El-Sayed, 2019). In addition, β-sitosterol has anxiolytic, and sedative, analgesic, anti-hypercholesterolemic, anti-inflammatory, anti-microbial, hypolipidemic, and hepatoprotective properties (Rather and Bhagat, 2020; Salehi et al., 2020).

#### 1.1.5 Volatile oils

Regarding compound types, ginseng volatile oil mainly contains monoterpenes, alkanes, esters, and sesquiterpenes. Among them, sesquiterpenoids are the characteristic components of ginseng volatile oil, which are further divided into sesquiterpenes (such as β-panasinsene, α-gurjunene, germacrene, β-gurjunene, β-elemene, β-caryophyllene, β-neoclovene, etc., formula C15H24, m/z 204) and sesquiterpene oxygenates (mainly alcohols such as spathulenol, PNN, globulol, α-cadinol, with molecular formulae C15H24O and C15H26O, m/z 220 and 222).

Sesquiterpenoids are the characteristic components of volatile ginseng oil. Reportedly, sesquiterpenoids have a wide range of biological activities, with various pharmacological effects, such as antitumor (Ham et al., 2019), anti-inflammatory (Chen et al., 2018), and antioxidant (Kang et al., 2013) effects. The sesquiterpene effective active monomer β-elemene is a class II non-cytotoxic antitumor drug in China and is used clinically for the treatment of lung, liver, and breast cancers, among others.

#### 1.1.6 Organic acids

Common organic acids in ginseng include citric, cinnamic, fumaric, maleic and salicylic acids. Modern studies have demonstrated that some organic acids in Chinese medicine also have biological activities, which may become an important direction for future research (Tang et al., 2012). Organic acids in ginseng, such as cinnamic acid, bind to various therapeutic targets and have broad-spectrum biological properties, including antibacterial, anti-viral, and anticancer activities. Mechanistically, cinnamic acid can inhibit microtubulin, histone deacetylase, NF-κB, adenosine 5′-monophosphate-activated protein kinase (AMPK) signaling, matrix metalloproteinase, and epidermal growth factor receptor in cancer cells (Feng et al., 2022).

#### 1.1.7 Proteins

Ginseng proteins (GP) are chemically active components present in the roots and leaves of ginseng plants. The protein content varies among different regions and species of ginseng depending on the origin of ginseng, with large differences in ginseng protein expression between high-latitude and low-latitude regions and smaller differences in ginseng protein expression between regions of the same latitude. GP are classified into RNA-like enzyme proteins, ribonucleases, saponin synthesis-related enzymes, chitin-like proteins, and xylanases, according to their functions, which are related to ginseng antifungal, anti-viral, and saponin synthesis, affecting cell proliferation and transcriptional activities. After extraction and purification, water-soluble GP have antioxidant, anti-radiation, immune boosting, hypoxia tolerance, and anti-fatigue effects. Also, they have memory improvement, anti-fatigue, anti-aging, tumor suppression, neuroprotection, and immune-boosting effects, which provide new ideas for herbal health care, beauty, and drug development (Li et al., 2022a).

#### 1.1.8 Ginseng polypeptides

Ginseng polypeptides consist of amino acids, which are precursor molecules of proteins that are easily absorbed by the body and have antibacterial, immune-enhancing, hypoglycemic, memory-improving, and hypoxia-resistant properties. They can also be used as a quality marker for different years of ginseng and have broad applications in the quality control of medicinal plants (Li G et al., 2022).

## 2 Anti-tumor mechanism of action of the active ingredients of ginseng

### 2.1 Inhibition of tumor cell proliferation

GS are the main active ingredients of ginseng, responsible for its main physiological activities and its major antitumor effects. For example, GRh2, Rg3, Rh4, Rk3, Rd, and C-K have good inhibitory effects on the proliferation of tumor cells in gastric, liver, colorectal and pancreatic cancers.

In gastric cancer cells, GRh2 and GRd inhibited the proliferation of gastric SGC-7901 cells in a dose-dependent manner (Han et al., 2020; Tian et al., 2020). GRh2 combined with PPD significantly inhibited proliferation and induced cytoplasmic vacuolization in gastric cancer HGC-27 cells by upregulating the expression of LC3II and p62, leading to mitochondrial damage, lysosomal dysfunction, and autophagic flow blockage (Qian et al., 2016). In HepG2 hepatocellular carcinoma (HCC) cells, GRh2 could exert an anticancer effert by activating glycogen synthase kinase GSK 3β and downregulating β-linked protein (Shi et al., 2016). In addition, GRh2 can specifically inhibit the proliferation of HepG2 (Zhang J et al., 2019) and Hep3B hepatoma cells by reducing H3K27me3 modification at the promoter of the CDKN2A-2B gene cluster site, promoting the transcription of tumor suppressor P14, P15, and P16 genes, and reducing EZH2 expression (Li et al., 2017). Zhang et al. observed that C-K downregulated Bclaf1 expression, inhibited the HIF-1α-mediated glycolytic pathway, and suppressed the proliferation of hepatoma cells (Bel-7404 and Huh7) (Zhang R et al., 2020). PPD has antioxidant, anti-inflammatory, and antitumor effects. In addition, it had the best inhibitory effect on HCC cell lines, inhibited the proliferation of HepG2 and PLC/PRF/5 hepatocytes in a dose-dependent manner, and suppressed the malignant progression of HCC (Yang L et al., 2019). PDZ-binding kinase/T-LAK cell-derived protein kinase (PBK/TOPK) is a cancer-testis antigen family member whose expression in normal tissues is only present in the testis and embryonic tissues and proliferating brain neural stem cells. It is highly expressed in various malignant tumors, such as colorectal, lung, and bile duct cancers, and is closely associated with the malignant biological behavior of cancer (He et al., 2010). In their study, Yang et al. observed that GRh2 inhibited the activity of PBK/TOPK and extracellular regulatory protein kinase 1/2 (ERK1/2) and (H3) phosphorylation levels to inhibit the proliferation of human HCT116 colorectal cancer cells (Yang et al., 2016).

The aberrant expression of PTEN, a classical oncoprotein, interferes with cell proliferation and apoptosis. Meanwhile, PTEN is a key regulatory protein of the PI3K/AKT signaling pathway and the two proteins are negatively correlated. Yang et al. observed that GRg3 regulates the PTEN/β-PI3K/AKT pathway, upregulates PTEN and P53 expression, downregulates p-pi3k and AKT expression, inhibits cell proliferation, and suppresses the malignant development of gastric cancer SGC-7901 (Yang L et al., 2020). C/EBPβ is a leucine zipper transcription factor that plays a crucial role in cell proliferation, differentiation and tumorigenesis. Numerous studies have shown that C/EBPβ plays a pro-cancer role in various cancers. Recently, C/EBPβ has been implicated in the regulation of human colorectal carcinogenesis (Zhang F et al., 2015). Yang et al. observed that GRg3 inhibited the reverse transcriptional activation of C/EBPβ, while the association of C/EBPβ with the NF-κB p65 subunit was reduced, and NF-κB activation was inhibited. Furthermore, GRg3 reportedly inhibited the proliferation of colorectal tumors HCT116, HT29, and SW480 cells partly through downregulation of the C/EBPβ/NF-κB signaling pathway and significantly inhibited the growth of xenografts in nude mice after 3 weeks of intraperitoneal injection of GRg3 (Yang et al., 2017). In addition, GRh3 inhibited the proliferation of colorectal cancer SW1116 cells in a dose- and time-dependent manner (Cong et al., 2020).

TGF-β, a molecule with transforming cellular properties, is an important activation signal for cellular functions and is important in cell differentiation, tissue repair, and immunosuppression. Smad 2/3, a TGF-β receptor-dependent activating transcriptional molecule regulated by TGF-β signaling and activates internal transcriptional activation in endothelial cells, thereby promoting cell proliferation. Jiang et al. observed that GRh4 exhibits strong anti-gastric cancer effects both *in vitro* and *in vivo*. Moreover, GRh4 was significantly inhibited GC cell HGC-27 and BGC-823 proliferation and colony formation through the six1-dependent TGF-β/Smad2/3 signaling pathway (Jiang et al., 2022). Liu et al. observed that in esophageal cancer cells, GRk3 triggered G1 phase blockade and activated apoptosis and autophagy by blocking the PI3K/Akt/mTOR pathway, thereby inhibiting the proliferation of Eca109 and KYSE150 cells, exerting anti-esophageal cancer activity *in vitro* and *in vivo* (Liu et al., 2019).

The JAK2/STAT3 signaling pathway is a signaling pathway widely shared by cytokines such as lymphokines and adipokines that mediate cell proliferation, apoptosis, and differentiation. For example, Fan et al. demonstrated that ginseng diol inhibited proliferation and induced apoptosis of pancreatic cancer cell lines PANC-1 and Patu8988 through the JAK2/STAT3 signaling pathway in a dose-dependent manner, limiting the malignant progression of pancreatic cancer (Fan et al., 2021).

GPS exhibits antitumor effects mainly through two mechanisms: direct inhibition of tumor cell proliferation and indirect demonstration of antitumor effects by improving the immune function of the body (Zhao, 2020). Cheng et al. divided GPS into neutral and acidic fractions, where the acidic group WGPA, mainly enriched in pectin and AG, exhibited significant inhibitory effects on cells and pectin-mediated antiproliferative activity (Cheng et al., 2011). GP can be hydrolyzed by pepsin, pancreatic enzymes, and dual enzymes, and enzymatic digestion produces mainly low molecular weight proteins, and small amounts of amino acids. Li et al. observed that total ginseng protein had the best inhibitory effect on Hep-2 cells *in vitro*, and its inhibition rate increased with the increasing protein concentration (Li et al., 2016a). Ren et al. observed that GP was induced through the mitochondrial pathway, blocking cell mitosis and inhibiting the ability of apoptosis, thus inhibiting the proliferation of cancer cells (Ren, 2019). Cheng et al. observed that GP enhanced the effect of H2O2-induced cancer cell damage, thereby inhibiting cell proliferation (Cheng et al., 2016). Ginseng pectin enriched with the HG structural domain had significant antiproliferative and cell cycle arrest effects on colon cancer cells in the G2/M phase, whereas HG-rich, RG-I, and HG-only pectin had significant inhibitory effects on the proliferation of liver cancer cells (Luo et al., 2017).

High concentrations of PNT (80 μmol/L) can regulate HCC HepG2 cytochrome P3A4 (CYP3A4) expression through constitutive androstane receptor (CAR), The effects of CAR and pregnane X receptor (PXR) have an interactive dialogue, and after regulation, CAR can inhibit the PXR-CYP3A4 pathway (Hu, 2018; Hu Q et al., 2019). PNT triol upregulationes CYP3A4 in human HCC HepG2 cells through the nuclear receptor PXR and upregulates PXR and CYP3A4 mRNA and protein expression after 24 h of intervention (Wu, 2017; Wu et al., 2019). In both human HCC HepG2 and Huh-7 cells, PNT upregulated the expression of the target gene CYP3A4 by attenuating the binding of PXR to HSP90α and promoting PXR-RXRα binding. At high concentrations, CAR was involved in the regulation of CYP3A4 through a similar mechanism. Moreover, during the upregulation of PXR-CYP3A4 by PNT triol, CAR can interactively inhibit PXR by counteracting its binding to RXRα (Zhang, 2021).

β-sitosterol inhibits cell growth by inducing apoptosis in human gastric cancer SGC-7901 (Zhao et al., 2009). β-sitosterol can mediate the AMPK/PTEN/Hsp90 pathway to inhibit the growth of human gastric cancer AGS cells *in vitro* and *in vivo* (Shin et al., 2018). Moreover, it also inhibits the proliferation of human colon cancer (HT-29) cells by stimulating the sphingolipid cycle (Khan et al., 2022). Cell nuclear antigen (PCNA) is an indicator of cell proliferative activity. In colon cancer, β-sitosterol decreases the expression of proliferating cell nuclear antigen (PCNA) (Sharmila and Sindhu, 2017). Shin et al. observed that the antitumor effects of β-sitosterol are mediated by the AMPK/PTEN/HSP90 axis in AGS human gastric adenocarcinoma cells and xenograft mouse models (Shin et al., 2018). Wang et al. observed that β-sitosterol inhibited the proliferation of colorectal cancer cells by regulating the reactive oxygen species (ROS)/AMPK/mTOR pathway to inhibit colorectal cancer cell proliferation (Wang G et al., 2022).

### 2.2 Induction of tumor cell cycle arrest

The ability of cells to complete the proliferation process is closely related to the cell cycle. Cell cycle regulation requires the cooperation of several extracellular and intracellular signals. However, in the absence of appropriate signals, cells will be unable to move to the next stage, a phenomenon called cell cycle arrest. In addition, cell cycle blocks help to maintain genetic stability, and cell cycle-regulated gene mutations play an important role in tumorigenesis.

Several studies have shown that GS can induce cell cycle blockade in various tumor cells to inhibit growth and exert antitumor effects. For example, GRh2 induced cycle arrest in the G0/G1 phase in gastric cancer cells SGC-7901 (Qian et al., 2016), HCC cells HepG2, Hep3B (Li et al., 2017), and colorectal cancer cells (LoVo/5-fluorouracil (5-FU) and HCT-8/5-FU) (Liu G et al., 2018), and inhibit tumor growth. Deng et al. observed that Rh4 inhibited PD-L1 growth by suppressing aerobic glycolysis and regulating the AKT/mTOR pathway to inhibit PD-L1 expression and induce G1 phase arrest to inhibit growth, thus exerting anti-esophageal cancer effects (Deng M et al., 2020).

GRg3, GRg5, Rd, and Re can induce cell cycle arrest in gastric cancer. For example, Zhang et al. obseved that GRg5 can increase the expression of P21 by reducing the production of the CCNG1/CDK 2/PCNA complex, causing cell cycle arrest in the S phase, and GRg5 can also regulate the cell cycle by inhibiting Notch1 protein expression, thus inhibiting the progression of AGS and MKN-45 in gastric cancer cells (Zhao et al., 2020); Liu et al. obseved that GRg5 can regulate the ROS-mediated MAPK pathway to induce gastric cancer cells SGC-7901 and BGC-823 to block the G2/M phase (Liu and Fan, 2019). Yang et al. obseved that Rg3 suppressed the malignant development of gastric cancer SGC-7901 by regulating the PTEN/β-PI3K/AKT pathway, upregulating PTEN and P53 expression and downregulating p-pi3k and AKT expression to block the cell cycle in the G1 phase (Yang M et al., 2020); In addition, GS-Rd induced cell cycle arrest in the G0/G1 phase in gastric cancer cells SGC-7901 and MKN-45 by downregulating cell cycle protein D1 (Tian et al., 2020). Jang et al. demonstrated that the heat treatment products GRg2, GRg6, and GF4 of ginsenoside Re inhibited the phosphorylation of CDK2 at Thr160 by upregulating p21 levels leading to an anticancer effect by blocking the AGS cell cycle in the S phase in gastric cancer cells (Jang et al., 2014).

PND is a polyacetylene compound isolated from ginseng, and its inhibitory effect of PNN on tumor cell proliferation is mediated by the impairment of cell cycle protein E mRNA levels and cell cycle transition from G1 to S-phase arrest (Yang Q et al., 2020). PNT significantly inhibited cell proliferation, induced G2/M cell cycle arrest in various tumor cell lines in a time- and dose-dependent manner, and delayed DNA synthesis in the human colon cancer cell line SNU-C2A (Kim et al., 2002). Furthermore, PND inhibited the proliferation of HepG2 HCC and led to morphological and ultrastructural changes in HepG2 cells, resembling the more mature hepatocyte form. PND blocked the transition from G1 to S phase in the HepG2 HCC cycle. Moreover, it significantly decreased the secretion of methemoglobin and the activity of γ-glutamyltransferase. In addition, PND increased the content of p21 mRNA levels but decreased Id-1 and Id-2 mRNA levels. Similarly, PND increased the protein levels of p21, pRb, and hypophosphorylated pRb in a dose-dependent manner (Guo et al., 2009).

Soysterol can induce apoptosis in HCC by upregulating the expression of the proapoptotic gene p53, which is also an important gene involved in the development of colorectal cancer and is closely related to the colorectal cancer-related signaling pathway PI3K/Akt. p53 can play an anti-tumor role by blocking the G1/G0 phase of cells and maintaining normal cell proliferation, which plays an important role in tumor evolution (Qu L et al., 2021). β-sitosterol effectively inhibits the proliferation and cell cycle arrest of AGS in human gastric cancer cells by activating the p53 signaling pathway and regulating apoptosis and cell cycle through upregulation of cleaved caspase-3 and cleaved PARP-induced apoptosis in gastric cancer AGS cells (Zhong et al., 2022).

Wang et al. observed that β-elemene induces cell cycle arrest in the G2/M phase by regulating the ROS/AMPK/mTOR pathway (Wang X et al., 2022).

### 2.3 Induction of apoptosis in tumor cells

Apoptosis is an autonomous and orderly cell death controlled by genes, which is the results from the co-regulation of multiple genes. Apoptosis can be activated by a variety of cellular signals, such as an imbalance in calcium homeostasis, oxidative damage, mitochondrial damage, toxins, growth factors, and hormonal stimulation. Classical apoptotic mechanisms can be divided into endogenous and exogenous signaling pathways. Endogenous apoptotic signaling usually activates the mitochondrial pathway. The exogenous death receptor pathway begins with the binding of specific death receptors to their ligands. Important factors mediating apoptosis include the Bcl2 family, caspases, death receptors (DRs), inhibitory apoptotic proteins (IAPs), and mitochondria-mediated apoptosis regulators.

Several studies have shown that GRh2, GRg3, and GC-K can induce apoptosis and inhibit tumor progression by upregulating pro-apoptotic genes. Liu et al. observed that GRh2 induced cycle arrest and apoptosis in drug-resistant colorectal cancer cells (LoVo/5-FU and HCT-8/5-FU) (Liu T et al., 2018). Zhang et al. observed that GRh2 induced apoptosis by activating the p53 pathway, upregulating Bax expression, decreasing Bcl2 expression, and inducing apoptosis in HCT116 and SW480 colorectal cancer cells (Zhang H et al., 2022). In pancreatic cancer cells, GRh2 also induces apoptosis in Bxpc-3 cells by upregulating Bax, caspase-3, and caspase-9, and downregulating Bcl-2, survivin, cyclin D1, MMP-2, and MMP-9 (Li et al., 2021a). Qian et al. observed that GRh2, by upregulating Bax and downregulating Bcl-2, induced apoptosis in gastric cancer cells SGC-7901 cells (Qian et al., 2016).

In hepatocellular carcinoma, Zhang et al. observed that GRh2 significantly increased apoptosis in HePG2 cells, which was associated with increased expression levels of caspase-3, caspase-6 and poly ADP-ribose polymerase proteins (Phi et al., 2019). Hu et al. observed that GRg3 induced apoptosis, initialized tumor apoptosis progression, and prolonged *in situ* HCC cell Hep1-6 model survival (Hu S et al., 2019). *Helicobacter pylori* cytotoxin-associated antigen A plays an important role in gastric cancer development. Rockwell glycosylation plays an important role in cancer biology and rockwell glycosyltransferase IV (FUT4) is an essential enzyme that catalyzes the synthesis of Lewy’s oligosaccharides and is regulated by the specificity protein 1 (SP1) and heat shock factor protein 1 (HSF1) transcription factors. Aziz et al., in their study, observed that GRg3 was significantly induced in the apoptosis of SGC-7901 cells; GRg3 significantly increased the expression of proapoptotic proteins and triggered the activation of caspase-3, caspase-8, caspase-9, and PARP; subsequently, it inhibited the development of gastric cancer by upregulating SP1 and downregulating HSF1 and suppressing FUT4 expression (Aziz et al., 2016). GRg3 also upregulated PTEN and P53 expression by regulating the PTEN/β-PI3K/AKT pathway, down-regulating p-pi3k and AKT expression, promoting apoptosis (Yang L et al., 2020). The sodium++/H++ exchanger 1 (NHE1) plays a crucial role in the development and progression of HCC and is a promising target for HCC treatment. Previous studies have shown that EGF significantly upregulates NHE1 expression and increases ERK1/2 and HIF-1α expression. Li et al. observed that after GRg3 treatment, NHE1 expression could be reduced by the overall inhibition of the EGF-EGFR-er k1/2-HIF-1 signaling axis, promoting apoptosis and, thus, inhibiting the malignant progression of HCC (Li et al., 2018).

In colorectal cancer cells, GRg3 can induce apoptosis in the colon cancer cell line HT-29 by activating the AMP-activated proteinase signaling pathway (Liu Z et al., 2021); GRg3(s) induces apoptosis in cancer stem cells by activating the caspase-3 and, caspase-9 pathways (Han et al., 2012); Lee et al. observed that GRg3 significantly inhibited NF-KB activity, activated caspase-3, and promoted the release of nitric oxide from the vascular endothelium in colon cancer cell lines, thereby inducing apoptosis in colon cancer cells (Lee et al., 2009). Zhang et al. investigated the effect of GRg3 on gallbladder cancer and showed that ginsenoside GRg3 induces apoptosis in gallbladder cancer cells by activating the p53 pathway (Zhang X et al., 2015).

C-K, a major intestinal microbial metabolite of Rb1, induces apoptosis in many cancer cells and has strong chemopreventive potential. Wang et al. confirmed the interaction between membrane-linked proteins A2 and C-K using molecular docking and thermal displacement assays. C-K blocked the interaction between membrane-linked protein A2 and the NF-кB p50 subunit and its nuclear co-localization, inhibited NF-кB activation, upregulated caspase-9 and caspase-3 expressions, and promoted apoptosis (Wang X et al., 2019). Liu et al. observed that C-K upregulates p21, a downstream target of RUNX3, arrests the cell cycle in the G0/G1 phase, and induces apoptosis (Liu G et al., 2022). Reportedly, C-K induced apoptosis by inhibiting caspase and p53-dependent LGR5 expression in HCT116 colorectal cells (Pak et al., 2020) and blocking HT-29 colorectal cells from entering the G1 phase, and C-K showed significant anti-inflammatory effects even at low concentrations (Yao et al., 2018).

Carbohydrate metabolism is the main mechanism through which organisms obtain energy. The anaerobic oxidation of sugar is known as glycolysis and is one of the main pathways for the oxidative breakdown of sugars. Generally, tumor cells undergo metabolic changes differently from normal cells—they can adapt to the altered metabolic environment and promote tumor progression by switching between glycolysis and oxidative phosphorylation. Shin et al. observed that C-K could promote tumor progression by inhibiting glycolysis and AKT/mTOR/c-Myc signaling and its downstream factors, hexokinase 2 and pyruvate kinase isozyme M2, inducing apoptosis in HepG2 and Huh7 HCC cells (Shin et al., 2021). Zhang et al. observed that C-K inhibited the growth and colony formation of hepatoma cells HepG2 and SMMC-7721 by downregulating p-STAT3 level, reducing the DNA binding ability of STAT3, and inducing endoplasmic reticulum stress (ERS) and apoptosis (Zhang et al., 2018).

Other active components of GS can also induce apoptosi; for example, GRh3 induces apoptosis in colorectal cancer SW1116 cells by upregulating the level of caspase3 expression (Cong et al., 2020). In gastric cancer, Tian et al. observed that GS-Rd promoted the pro-apoptotic process in gastric cancer cells SGC-7901 and MKN-45 by increasing the expression of caspase-3, caspase-9, and the Bax/Bcl-2 ratio and inducing cell cycle arrest by downregulating cell cycle protein D1 (Tian et al., 2020). Ginsenoside F2 (GF2), a pro-ginsenoside diol-type saponin, inhibits various tumor cells. It inhibits the development of the human gastric cancer cells line SGC7901 by regulating the ribosomal protein-p53 signaling pathway and the Bcl-xl/Beclin-1 pathway (Mao et al., 2016), In addition, GF2 can also induce the apoptosis of gastric cancer cell SGC-7901 by causing the accumulation of ROS and activating the ASK-1/JNK signaling pathway (Mao et al., 2016). Lu et al. observed that Rk1 could inhibit the progression of HepG2 HCC by inhibiting the ERK/c-Myc pathway, downregulating the expression of glutaminase GLS1, reducing the production of glutathione GSH, stimulating ROS accumulation, and inducing apoptosis (Lu et al., 2022). The MAPK pathway to induces apoptosis in SGC-7901 and BGC-823 human gastric cancers (Liu and Fan, 2019). GRg5, in combination with Rk1, can also regulate the MAPK/NF-κB pathway, target apoptotic and anti-apoptotic genes, promote the endogenous apoptotic pathway, and induce apoptosis in HCC cells MHCC-97H (Chen C et al., 2021).

In addition, one study reported that ginseng diol induced apoptosis in the pancreatic cancer cell lines PANC-1 and Patu8988 through the JAK2/STAT3 signaling pathway, limiting the progression of pancreatic cancer (Fan et al., 2021). Ding et al. observed that Sijunzi tang, with GS as one of the main components, downregulates the expression of VEGFA, iNOS, COX-2 and Bax/Bcl2, regulates the PI3K/AKT pathway, and induces apoptosis to treat gastric cancer cells NUGC-4 (Ding et al., 2022). (24r)-Ginsenoside HQ (R-PHQ) and (24s)-Ginsenoside HQ (S-PHQ) are the main metabolites of (20s)-Ginsenoside Rh₂ (GRh₂) *in vivo*. Qi et al. observed that the combination of GRh₂, R-PHQ, and S-PHQ significantly increased the expression of Bax and inhibited Bcl-2 to induce apoptosis in HCC H22 cells (Qi et al., 2019).

ROS-mediated apoptotic pathway plays an important role in the cell death process. ROS at higher ROS levels can lead to cellular senescence or death. Ginsenoside epoxynol induces apoptosis by increasing intracellular calcium levels, activating JNK and p38 MAPK and NADPH oxidase-dependent ROS production (Kim et al., 2011), and activating EGFR, the CAMKII-TAK1-p38/JNK pathway and ERS (Kim et al., 2016). Soysterol induces apoptosis in the human HCC cells line SMMC-7721 by ROS oxidation, a cascade reaction that increases calcium ion concentration and blocks the S and G2/M phases of the cell cycle (Li et al., 2012). Ditty et al. observed that β-sitosterol induces ROS accumulation in human HCC HepG2 cells through an endogenous pathway, leading to membrane damage and mitochondrial toxicity, inducing cytochrome C release from the mitochondria, and enhancing the protein expression of caspase-3 and cleaved caspase-3 (Ditty and Ezhilarasan, 2021). Dihydroxy cinnamic acid (DHCA, commonly known as caffeic acid), a cinnamic acid derivative, decreases HDAC activity, induces caspase-3 mediated apoptosis by generating ROS, blocking cells in the S and G2/M phases to induce cancer cell death, abd inhibiting apoptosis in colon cancer cells (Anantharaju et al., 2017). The thioredoxin-thioredoxin reductase (Trx/TrxR) system plays a key role in cancer and is a novel drug target for regulating cancer cell stress. Trx1 and TrxR1 expression is enhanced in many cancer cells to control ROS homeostasis, promote cell growth, and foster resistance to apoptosis (Shen et al., 2019). β-sitosterol limits the protein expression of Trx/TrxR1, which triggers the accumulation and activation of ROS during apoptotic cell death (Rajavel et al., 2018).

In human colon cancer cells HT116, β-sitosterol reduced the expression of Bcl-2 and inhibited the apoptosis of protein-1 (cIAP1) while inducing the activation of BAX and cytochrome C. It also reduced tumor size in a dose-dependent manner and induced apoptosis in HCT116 colorectal cancer cells both *in vitro* and *in vivo* through the EGFR/Akt pathway (Kawk et al., 2021). β-sitosterol also induces apoptosis through a cystathionine-dependent pathway (Raj et al., 2022). Wang et al. compared two ginseng berry polysaccharide preparations and observed that GPS had a significant inhibitory effect on inflammation-associated colon cancer, inhibiting T-cell differentiation and promoting apoptosis (Wang C et al., 2020).

Tang et al. observed that stigmasterol could effectively inhibit the proliferation of gastric cancer cells MGC-803 in a dos-dependment manner and also induced apoptosis in gastric cancer MGC-803 cells by regulating the apoptotic signaling pathway, activating caspase3 and caspase9, upregulating Fas and Bax gene expression, and downregulating Bcl-2 protein (Tang, 2021). The inhibition of SMMC-7721 in HCC cells *in vitro* is dose- and time-dependent and can significantly downregulate several oncogenes such as fos, myc, and ras and upregulate the expression of oncogenes represented by NF-2 and the phosphokinase Map2k6 (Zhang et al., 2007). Kim YS et al. demonstrated (Kim et al., 2014) that dousterol can induce apoptosis in HepG2 cells by upregulating pro-apoptotic gene expression (Bax protein and p53 gene), downregulating anti-apoptotic gene Bcl-2 expression, activating pro-apoptotic caspase-8, caspase-9 protein expression, and by damaging cellular DNA, and can be used as a potential anti-cancer drug for liver cancer treatment. NDEYet et al. observed that dousterol upregulated p27 expression and downregulated jab1 in human gallbladder cancer cells, thereby regulating the mitochondrial apoptotic signaling pathway, activating caspase-3, and inducing apoptosis (Pandey et al., 2019). Soysterol can also induce apoptosis and protective autophagy in gastric cancer cells by inhibiting the Akt/mTOR pathway (Zhao et al., 2021) and may be a potential anticancer drug for the treatment of gastric cancer.

Alkaloid-cinnamic acid hybrids exhibited potent effects on all three human tumor cell lines (HeLa, HepG2, and A549), induced apoptosis in HepG2 cell lines, altered mitochondrial membrane potential, and produced ROS, leading to apoptosis in HepG2 cells (Shang et al., 2020). These compounds could be promising lead compounds for further development as antitumor agents through structural modifications.

Wang et al. observed that β-eugenol induced apoptosis by regulating the ROS/AMPK/mTOR pathway and upregulating the expression of the proapoptotic genes caspase-3, caspase-9, and PARP protein (Wang G et al., 2022). Bomfim et al. observed that β-eucalyptol induced caspase-mediated apoptosis in human HCC HepG2 cells, decreased cell proliferation and induced tumor cell death (Bomfim et al., 2013). Cholangiocarcinoma (CCA), an epithelial malignancy of the bile ducts, is a aggressive with a poor prognosis and unsatisfactory response to chemotherapy with acquired drug resistance. nAD(P)H-quinone oxidoreductase 1 (NQO1) is an antioxidant and detoxifying enzyme that plays an important role in drug resistance and the proliferation of several cancer cells. Srijiwangsa et al. observed that β-eucalyptol significantly inhibited KKU cell proliferation in a concentration-dependent manner, significantly inhibiting the expression of NQO1 enzyme activity in KKU-100 cells from activating relevant apoptotic pathways and promoting apoptosis (Srijiwangsa et al., 2018).

### 2.4 Enhancement of tumor cell immunity

The tumor microenvironment (TME) is a complex system that includes tumor cells and multiple interdependent immune and stromal cell populations such as T, B, natural killer, and myeloid cells (Hamilton et al., 2022). These immune cells exhibit a broad immune diversity and play a crucial synergistic role in tumor control (Laumont et al., 2022). Therefore, it is important to improve the immune capacity of tumor cells and stimulate strong and durable anti-tumor immunity.

GRg3 increases the expression of molecules such as HLA-DR, HLA- ABC, and CD56, promotes the proliferation of splenic lymphocytes, causes T helper 1 (Th1)/T helper 2 (Th2) and CD4+/CD8+ cells to drift in the direction of immune enhancement, and enhances the immune function of peripheral lymphocytes (Zhang et al., 2004). Animal experiments showed that serum levels of interleukin (IL)-2 and In- terferon γ (IFN-γ) were significantly increased in HCC H22 cell-bearing mice treated with GRg3, and stimulated ConA, induced lymphocyte proliferation, and enhanced immunity (Wu et al., 2014). Also, GRg3 also increases the activity of NK cells and positively regulates immune function (Lin et al., 2002).

NK cells are innate immune cells with potent cytotoxic and cytokine production capabilities, which play a central role in cancer immune surveillance (Mylod et al., 2022). 20(R)-Rg3 increases the expression of NK activation receptors NKp44, NKp46, NKp30, and NK cell degranulation marker CD107a through activation of the MAPK/ERK pathway, thereby enhancing the killing activity of NK cells against tumor cells (Lee et al., 2022). Xia et al. observed that GRh2 could regulate immune factors (e.g., IL4, IL6, CD3, CD45, and INF-γ) to enhance splenic immunity and increase the number of NK cells, which are important in tumor prevention and treatment (Xia et al., 2020). In addition to digestive system tumors, in MCF7 breast cells, GRh2 downregulates CASP1, INSL5, and OR52A1 and upregulates the expression of CLINT1, ST3GAL4, and C1orf198, induces an immune response and epigenetic methylation changes in tumors, enhances immunogenicity, and inhibits the growth of cancer cells (Lee et al., 2018).

Li et al. observed that GFP1 could inhibit tumor growth and lung metastasis *in vivo* by activating immune function, increasing the relative weights of the spleen and thymus, promoting splenic lymphocyte proliferation, and increasing splenic NK cell activity (Lee et al., 2019). Li et al. observed that GPS modulates the TME by stimulating immune cells (Li et al., 2021b). Li et al. observed that polysaccharides prolonged the survival of HCC H22-bearing mice and that the use of GPS enhanced the host defense response against tumors (Li et al., 2016b). In addition, GPS enhances macrophage and dendritic cell activity and phagocytosis and promotes cell maturation (Zhang X. Y. et al., 2017).

The Nrf2 signaling pathway is a classical anti-oxidative stress-related pathway that upregulates antioxidant defense mechanisms. β-sitosterol significantly reduces intracellular ROS levels, affects the Nrf2 pathway and acts as an antioxidant, which is beneficial in the treatment of cancer and complications caused by oxidative stress (Gangwar et al., 2021). β-sitosterol significantly hindered the expansion of transplantable tumors, protected lung parenchyma, and increased splenocyte proliferation and cytotoxic t lymphocyte activity in tumor-bearing mice, and enhanced host macrophage lysosomal activity, and antioxidant cellular activity, and inhibition of lipid peroxidation. The antitumor effect of β-sitosterol is related to its immunomodulatory activity (Boubaker et al., 2018).

β-sitosterol (CN3) blocks the secretion of Th2 cytokines (IL-4 and IL-10). However, there was no effect on the secretion of Th1 cytokines (IL-2 and IFN-γ), suggesting that β-sitosterol treatment selectively inhibited Th2 activity and promoted Th1 bias. CN3 was also found to significantly decreased the proliferation of T helper cells (CD4CD25) and cytotoxic T cells (CD8CD25) after ConA-induced T-cell activation (Le et al., 2017). β-sitosterol has immunomodulatory effects and has potential for development as an immunotherapeutic agent.

Soysterols are natural sterols with defined immunomodulatory properties that attenuate the innate and adaptive immune responses (Antwi et al., 2018), have potential anti-inflammatory effects, and can inhibit pro-inflammatory and matrix degradation mediators (Liang et al., 2020). In addition, Sabeva et al. reported that soysterols stimulate lymphocyte proliferation in the blood. Proliferation is accompanied by the secretion of IL-2 and IFN-γ by Th1 cells, which enhances the lysis of NK cells and produces an immune response (Sabeva et al., 2011).

### 2.5 Inhibition of tumor invasion and metastasis

Invasion and migration of tumor cells are malignant behavior of tumor cells; invasion refers to the invasion or occupation of malignant tumors from the primary or secondary tumors to adjacent host tissues; metastasis refers to a recurrent multistep process in which the primary tumor spreads to distant organs. Tumor cells leave the primary tumor to invade the surrounding tissues, enter the blood or lymphatic vessels, and are transported to distant sites to recolonize in a new organ environment (Cuypers et al., 2022). Tumor metastasis is an important cause of cancer treatment failure and recurrence (Ishay-Ronen et al., 2019).

#### 2.5.1 Angiogenesis

Angiogenesis plays an important role in the growth and metastasis of tumors by providing essential nutrients and oxygen (Riabov et al., 2014). It has been shown that GRh2 inhibits angiogenesis by reducing the expression of JAM, CD31, vascular endothelial growth factor (VEGF), platelet-derived growth factor, and CNNM1 in cancer cells, thereby inhibiting tumor growth (Huang et al., 2019). (20S)-GRh2 inhibited tumor cell growth and peripheral angiogenesis by targeting membrane-linked protein A2 to inhibit STAT3/VEGF signaling (Wang B et al., 2021). GRg3 inhibits VEGF-induced angiogenesis through the PI3K/Akt pathway and its downstream signaling molecules in HCC cells (Hu Q et al., 2019) and endometrial carcinoma (Cao et al., 2017) and regulates the expression of downstream regulators p70S6K and HIF-1α (Zhang X. et al., 2017). In addition, GRg3 inhibits pancreatic cancer angiogenesis by downregulating VE-cadherin/Eph a2/MMP9/MMP2 expression (Liu Y et al., 2021). Nakhjavani M et al. observed that HCC treatment with GRg3 combined with arterial embolization significantly inhibited tumor angiogenesis, slowed tumor progression, and significantly reduced the rate of tumor metastasis (Nakhjavani et al., 2020).

Kangsamaksin et al. observed that soysterols significantly reduced tumor necrosis factor-α (TNF-α) transcript levels *in vitro* and disrupted tumor angiogenesis, and inhibited the growth of CCA xenografts *in vivo* (Kangsamaksin et al., 2017). Soysterols also stimulate cell death and inhibit cell migration and angiogenesis by activating the endoplasmic reticulum-mitochondrial axis (Bae et al., 2020).

#### 2.5.2 Epithelial-mesenchymal transition

Activation of epithelial-mesenchymal transition (EMT) is a key process in cancer cell metastasis, in which epithelial cells acquire mesenchymal cell characteristics, such as deficiency of epithelial cell markers (e.g., cytokeratins and E-cadherin) and upregulation of mesenchymal cell markers (e.g., N-cadherin, vimentin, and fibronectin) are upregulated (Banyard and Bielenberg, 2015). In addition, EMT leads to the loss of apical cell polarity in epithelial cells, reorganization of the cytoskeleton, and reprogramming of gene expression, thereby promoting an aggressive phenotype in cancer metastasis (Lamouille et al., 2014).

20(S)-GRh2 (SGRh2) up-regulates E-cadherin and down-regulates N-cadherin, vimentin, and EMT transcription factors (such as Smad-3, Snail-1, and Twist-1) and downregulates matrix metalloproteinases (MMP-2 and MMP-9), thereby inhibiting the metastasis of colorectal cancer (Yuan et al., 2020). PPD inhibited the expression of epithelial mesenchymal transformation markers in HepG2 and PLC/PRF/5 hepatocytes in a dose-dependent manner, increased the expression of E-cadherin, and decreased the expression of waveform proteins (Yang P et al., 2019). GRb2 significantly reduced the number of metastatic nodules in the liver, lungs, and kidneys of metastatic mice by downregulating the expression of stemness and EMT-related genes through the EGFR/SOX2 signaling axis (Phi et al., 2018).

Matrix metalloproteinases, which degrade most of the ECM and basement membrane protein components, are important molecules in a complex system that regulate tumor invasion and metastasis as well as proliferation, differentiation and cell death (Conlon and Murray, 2019). GRg5 inhibits the migration of MNK-45 cells by reducing MMP2 and MMP9 (Zhao et al., 2020). GRh2 inhibits the migratory ability of HepG2 HCC cells by recruiting HDAC, suppressing AP-1 transcription factors, and reducing MMP3 expression levels (Zhang K et al., 2019).

As tumor cells continue to multiply, the original oxygen or nutrient supply becomes inadequate, and this hypoxic environment drives tumor cells to upregulate growth factors and chemokines, thereby promoting tumor cell metastasis (Banyard and Bielenberg, 2015). GC-K impairs the metastatic potential of HCC cell lines under hypoxic conditions and inhibits hypoxia-induced or TNF-α-stimulated expression of the HIF-1α/NF-κB signaling pathway and as well as EMT markers in HCC cells (Zhang F et al., 2021). GRg3 inhibits the expression of HIF-1α and VEGF in the human gastric cancer cells line BGC823 and may affect the peritoneal implantation of gastric cancer metastasis by suppressing their expression (Li and Qu, 2019).

In pancreatic cancer metastasis, 20(S)-GRh2 effectively inhibited IL-6-induced signaling and STAT3 phosphorylation, MMP-1, -2, and -9 expression, suppressed migration and invasion of pancreatic cancer Bxpc3 cells (Li et al., 2021a), prevented degradation of the extracellular matrix and basement membrane, and inhibited EMT progression (Han et al., 2016). GRh2 also inhibited human pancreatic cancer cell Bxpc-3 migration by downregulating MMP-2 and MMP-9 (Li et al., 2021b).

ARHGAP9 belongs to the Rho GTPase family and regulates cell migration during cancer progression by promoting GTP hydrolysis through various downstream signaling pathways to inactivate small GTPases (Amin et al., 2016). ARHGAP9 knockdown enhanced tumor migration and invasion. GRg3 effectively inhibited the migration and invasion of HCC cells HepG2 and MHCC-97L and tumor growth in BABL/c nude mice by upregulating the protein expression of ARHGAP9 (Sun et al., 2019). In addition, ginsenosides regulate various signaling pathways, such as Notch-Hes1 and TGF-β/Smad2/3, to inhibit EMT in tumor cells (Zhao et al., 2020).

#### 2.5.3 Extracellular matrix

Neurociliary protein-1 (NRP1) is highly expressed in progressive gastric cancer and is associated with a poor prognosis. NRP1 interacts with fibrillin-1 (FN1) to promote the malignant progression of gastric cancer cells by affecting cell survival and migration through ECM remodeling. GRg3 downregulates NRP1 expression, blocks the interaction between NRP1 and FN1, and inhibits the malignant progression of gastric cancer MGC-803/MKN-28 (Wu et al., 2020).

#### 2.5.4 Cancer stem cells

Cancer stem cells are a major driver of recurrence in many cancers (Phi et al., 2019). 20(R)-GRg3 inhibits the tumor stem cell properties of HT29 and SW620 colorectal cancer cells through the SNAIL signaling axis, downregulates the expression of stemness genes and EMT markers in CRC cells and suppresses motility and EMT in colorectal cancer (CRC) cells (Phi et al., 2019). C-K effectively inhibits the proliferation and migration of mesenchymal stem cells to CCA cells, suppressed, Wnt/β-catenin signaling pathway activation, and inhibits the metastatic growth of CCA cells (Wang et al., 2015).

### 2.6 Tumor cell autophagy

Autophagy is a catabolic process that degrades cytoplasmic components and organelles in the lysosomes. There is growing evidence that autophagic signaling is closely related to oncogenic signaling. Selective targeting of autophagy for cancer treatment has attracted considerable attention (Ryan, 2011). Thus, autophagy may be an effective way to prevent tumor formation and progression (Mizushima et al., 2008). GRh2 promotes cellular autophagy by upregulating autophagy-related genes ATG5, ATG7, LC3B, beclin1, and the LC3II to LC3I ratio (Liu et al., 2015), and by inhibiting the PI3K/AKT/mTOR pathway (Zhuang et al., 2018; Li et al., 2019). Liu et al. observed that ginsenoside Rg5 induces autophagic processes in SGC-7901 and BGC-823 human gastric cancers by regulating the ROS-mediated MAPK pathway (Liu and Fan, 2019). Wang et al. observed that β-elemene induces a cellular autophagic response through the upregulation of LC3B and SQSTM1 expression (Guo et al., 2022).

### 2.7 Intestinal flora

The diversity of the intestinal bacterial community is closely related to human health, and intestinal microbes perform various physiological and biochemical functions. There is growing evidence that the gut microbiota plays a key role in the metabolism of nutrients and drugs, the absorption of dietary fats, and the regulation of immunity, physiology, metabolism, and health maintenance. In addition, gut microbes can affect multiple tissues and organs, and specific changes in the composition of the gut microbiota have been associated with various diseases; therefore, the gut microbiota is considered a potential target for the prevention and treatment of various diseases (Huang G et al., 2017).

Previous studies have shown that GS and GPS can be used to modulate the structure of the gut microbiome to treat various diseases. Chen et al. observed that Rk3 repaired intestinal barrier dysfunction by increasing the expression of tight junction proteins and reducing colonic inflammatory cytokine levels, oxidative stress, and macrophage infiltration. Rk3 effectively improves intestinal flora metabolic dysregulation by inhibiting the TLR4/NF-κB signaling pathway (Chen H et al., 2021). Huang et al. observed that Rb3/Rd improves intestinal epithelial cells by restoring the expression of E-cadherin and N-cadherin. By increasing anti-inflammatory cytokines and decreasing pro-inflammatory cytokines, the mucosal immune structure was remodeled and mucosal immunity was improved. In addition, Rb3/Rd significantly reduced the abundance of cancer cachexia-associated bacteria, such as *Dysgonomonas sp*. and *Helicobacter sp*., thus restoring the intestinal flora and intestinal microenvironment to exert anti-cancer effects [159]. Oral GPS combined with anti-PD-1-mAb can improve the therapeutic sensitivity of anti-PD-1-mAb in non-small-cell lung cancer (NSCLC) patients. This effect may be related to GPS-induced remodeling of the intestinal flora structure in chemotherapy non-responders, which leading to an increased abundance of metabolites such as short-chain fatty acids (scfa), along with the down-regulation of IDO activity. Thus, the immunosuppressive TME associated with NSCLC is altered, allowing for increased sensitivity to immunotherapy induced by GPS administration (Li et al., 2021a).

Sitosterol maintains a diverse microbial environment, resulting in beneficial metabolites, including those that promote apoptosis in tumor cells. Sitosterol decreases PI3K/Akt expression in colon cancer tissues, promotes Bad activation, decreases Bcl-xl expression, and enhances cytochrome C release, leading to caspase-9 and caspase-3 activation, PARP cleavage, and apoptosis. Moreover, the diversity of microbiota, particularly the phylum *Bacteroides* and the thick-walled phylum, was significantly reduced in the intestines of colon cancer-bearing mice, and sitosterol treatment reversed these changes. Sitosterol treatment increases SCFA levels in mouse fecal samples, leading to apoptosis of cancer cells *in vitro* (Ma H et al., 2019).

### 2.8 Epigenetic regulation

Epigenetic modifications in the host genome are critical for the adaptation of organisms to extreme conditions. DNA methylation, covalent modification of histones and interlinking of non-coding RNAs contribute to the cellular expression of epigenetic changes in the genome. Among the various factors involved in host epigenetic programming, non-coding RNAs (ncRNAs) such as microRNAs (miRNAs), long-stranded non-coding RNAs (lncRNAs), and cyclic RNAs, affect a various cellular processes, such as tumor immunity and cell differentiation (Roy et al., 2022).

GC-K significantly inhibits the growth of colorectal cancer cells by inhibiting the DNA expression and activity of methyltransferase 1, inducing demethylation of the RUNX3 promoter in HT-29 human colorectal cancer cells, causing RUNX3 mRNA and protein re-expression and localization in the nucleus (Salehi et al., 2020). GRg3 reduces the expression of p-AKT, p-pi3K, MMP2, and MMP9, decreases lncRNA HOTAIR expression, and inhibits the proliferation and invasion of SMMC-7721 and SK-Hep-1 HCC cells by regulating the PI3k/AKT pathway (Pu et al., 2021). GRg3 also inhibited the migration and invasion of colorectal adenocarcinoma Caco-2 cells by suppressing the expression of lncRNA CCAT1 (Li and Qi, 2019). lncRNAs HOTAIR, SP1, and PDK1 exhibit oncogenic effects in many tumors and are involved in cancer development and progression by mediating multiple signaling pathways. Wu et al. observed that β-elemene inhibits HCC cell proliferation by regulating lncRNA HOTAIR, SP1, PDK1, and their interactions (Wu J et al., 2022).

miRNA expression is essential for the regulation of complex genetic networks and cellular signaling cascades. In disease states, miRNAs can alter protein expression to play a central role in pathological cellular changes (Subramanian and Steer, 2019). Single miRNAs can direct entire cellular pathways and regulate a broad spectrum of targets (Gebert and Macrae, 2019). Thus, targeting miRNAs can regulate complex disease networks and interfere with most molecular pathological mechanisms (Diener et al., 2022). GRh2 increases the expression of miR-200b-5p, miR-224-3p, and miR-146a-5p and decreases the expression of miR-26b-3p and miR-29a-5p, effectively inhibiting the survival of HepG2 cells, promoting apoptosis, and suppressing colony formation in HCC cells (Chen et al., 2018). 20(S)-Rg3 mediates miR-532-3p/miR-324-5p to suppress PMK2 expression (Zhou et al., 2018; Cheng and Xing, 2019) and inhibits cell viability and the cell cycle in esophageal squamous cell carcinoma by regulating miR-324-5p-targeted PSME3, thereby promoting apoptosis (Jiang et al., 2021). Deng et al. observed that β-elemene inhibited lung metastasis (Deng X et al., 2020) and peritoneal metastasis (Deng et al., 2019) in gastric cancer cells SGC7901/ADR by regulating the miR-1323/Cbl-b/EGFR and FAK/Claudin-1 signaling pathways.

### 2.9 Derivatives

Pseudoginsenoside Rh2 (pseudo-G-Rh2) is a novel GRh2 derivative with proapoptotic effects in various malignancies. For example, pseudo-G-Rh2 significantly increased the expression of pro-apoptotic genes, increased the accumulation of autophagosomes and autolysosomes in HepG2 HCC cells, activated AMPK, and inhibited the PI3K/Akt/mTOR pathway in a concentration-dependent manner (Zhang J et al., 2021). Similarly, GRg3 and ursolic acid co-loaded liposomal UA + Rg3-LIP significantly reduced cell viability, promoted apoptosis, increased the ratio of G0/G1 phase cells, and slowed the *in vitro* drug release ability of HCC cells (Wang C et al., 2021).

In the context of gastric cancer context, 4-XL-PPD, a novel ginsenoside derivative, inhibits the viability of BGC-803 gastric cancer cells and produces ROS to inhibit migration and invasion-associated proteins (MMP-2, MMP-9, E-calmodulin, and CD34) and induce apoptosis (Wang Y et al., 2019). The ginsenoside metabolite M1 at a concentration of 100 μg/ml, was highly cytotoxic to MGC80-3 human gastric cancer cells (Li et al., 2013). 2-Pyrazine-PPD induces apoptosis in BGC-803 gastric cancer cells by activating the PERK/eIF-2α/ATF4 axis through the mitochondrial pathway and upregulating C/EBP-CHOP expression levels and ERS, thus exhibiting significant anticancer activity (De Wang et al., 2020). Ginsenoside derivative 6d induced apoptosis in HCT-116 colon cancer cells by regulating the MEK/ERK signaling and mitochondrial pathways, significantly upregulating the expression of Cyt-c and Cl-caspase-3/9/PARP, regulating the expression levels of p53, p21, cyclin B1, and CDK1, and inducing G2/M phase block (Ma et al., 2021).

The ginsenoside derivative Rh2-O has elevated immunomodulatory effects, enhancing the proliferative capacity and cytotoxicity of splenic lymphocytes through TLR4 and limiting cytokine secretion (e.g., IFN-γ, IL-2, and IL-4) in splenic lymphocytes (Wu et al., 2021). Among the 13 synthetic ginseng diol PD derivatives, number 3.12.13 showed significant inhibitory effects on the human liver cancer cell line, HepG-2, and human colon cancer cell line, HCT-116, and both showed low or no toxic effects on normal cells (Xiao et al., 2020), These derivatives can be used to develop new antiproliferative agents.

## 3 Combined use and reversal of drug resistance

Chemotherapy, as one of the main treatments for GI tumors, has remarkable therapeutic effects. As the number of chemotherapy cycles continues to increase, tumor cells become less sensitive to chemotherapeutic agents and develop chemoresistance, leading to tumor recurrence and metastasis. Ginseng is often used in combination with chemotherapeutic drugs to promote the effectiveness of chemotherapy and reduce toxicity. The risk of cancer recurrence was 50% lower in patients taking ginseng than in those who did not (Xu et al., 2021). Moreover, ginseng and its active ingredients combined with chemotherapy can enhance the effects of chemotherapy and reduce toxic side effects, which can significantly improve the survival rate and prolong the survival period of patients.

In colorectal cancer cells, Liu et al. (Liu G et al., 2018) observed that GRh2 downregulated the expression of drug-resistant genes such as multidrug resistance-associated protein 1 (MRP1), multiple drug resistance (MDR1), lung-resistance-related protein (LRP) and Glutathione S-transferase (GST), enhanced the cytotoxicity of 5-FU against drug-resistant colorectal cancer cells (LoVo/5-FU and HCT-8/5-FU), and effectively reversed 5-FU resistance of CRC cells. Furthermore, GRh2 reverses oxaliplatin resistance in colon cancer cells (Ma J et al., 2019) and adriamycin resistance in breast cancer MCF-7 cells by reducing the expression of P-glycoprotein (Zhao et al., 2018). The combination of GRh2 and L-OHP reversed oxaliplatin resistance in LoVo/L-OHP colon cancer cells by down-regulating the expression of P-gp and Bcl-2, and up-regulating the expression levels of Smad4, Bax, and caspase-3, and regulating the expression of drug resistance genes (Ma H et al., 2019). 20(S)-Rh2 is a secondary saponin of the ginseng diol group with enhanced anticancer activity. Han et al. demonstrated that a combination of adriamycin and 20(S)-Rh2 enhanced anticancer sensitivity and reduced resistance to adriamycin treatment in CRC cells (Han et al., 2016).

N6-methyladenosine (m6A) RNA methylation is commonly dysregulated in cancer, resulting in anticancer resistance. IGF2BP3 is a specific receptor for m6A in colon cancer, and IGF2BP3 promotes DNA replication and angiogenesis, contributing to cancer development. Paramasivam et al. observed that GRh2 reduces m6A RNA methylation in cancer through a KIF26B-SRF positive feedback loop and inhibits m6A autophagy-mediated anticancer drug resistance (Paramasivam and Priyadharsini, 2021).

The activation of P-gp as a classical mechanism of chemotherapy resistance has been associated with resistance to several chemotherapeutic agents, such as anthracyclines, vincristine, and paclitaxel (PTX) (Wu K et al., 2022). GRg3 is a P-gp inhibitor that can effectively reverse resistance to chemotherapy. Xu et al. observed that GRg3 could block drug efflux by inhibiting P-gp expression, blocking the binding site of P-gp, inhibiting cell membrane fluidity, and competing with anticancer drugs to bind to P-gp (Xu et al., 2021). Reportedly, GRg3 enhances the cytotoxicity of chemotherapy in gastric (Wang G et al., 2022) and colon cancers (Hong et al., 2020) by modulating the PI3K/AKT pathway, promoting apoptosis, and enhancing the chemosensitivity of both cisplatin and 5-FU, and reversing the chemoresistance of sorafenib and 5-FU. In addition, ginsenoside Rg3 can be combined with oxaliplatin for HCC (Lu et al., 2018) and oxaliplatin + 5-FU co-combination for colorectal cancer (Tang et al., 2018), both of which enhance its cytotoxicity and sensitivity to chemotherapy.

The oncogene PTEN plays important roles in cell growth, apoptosis, adhesion, migration, and infiltration. PTEN is an evaluation indicator for the prognosis of numerous tumors, and the study of its mechanism of action is important for tumors diagnosis and gene therapy (Ahmed et al., 2022). Lu et al. observed that sorafenib combined with 20(S)-Rg3 significantly reduced the viability of human HCC cell lines HepG2 and Huh7, promoted apoptosis, increased chemotherapy sensitivity, and reversed chemoresistance by modulating the PTEN/Akt signaling pathway. GRg3 also inhibited growth and promoted apoptosis of gemcitabine-resistant pancreatic cancer cells through upregulation of the PTEN signaling pathway (Zou et al., 2020).

In addition to GRh2 and GRg3, other active ginsenoside ingredients also enhance chemotherapy sensitivity and reverse drug resistance. Zheng et al. observed that ginsenoside Ro inhibited autophagosome-lysosome fusion through the ESR2-NCF1-ROS pathway, delayed checkpoint kinase 1 degradation, downregulated the DNA replication process, enhanced 5-Fu cytotoxicity, sensitized esophageal cancer cells to 5-FU chemotherapy, and led to delayed DNA repair and DNA damage accumulation (Zheng et al., 2016). C-K synergistically enhances the antitumor effects of 5-FU or adriamycin, induces apoptosis, and reduces survival genes and cytotoxicity in HCT116 colorectal cells (Pak et al., 2020). Sirtuin belongs to the mammalian silent information regulator 1 family, and studies have shown that overexpression of sirtuin 1 (SIRT1) is associated with resistance to chemotherapy (Xiong et al., 2017), which may promote proliferation and drug resistance in HCC cells by affecting the PI3K pathway and MRP1 expression (Ling et al., 2017).

ActD increased the expression and activity of SIRT1 in drug-resistant LS513 colon cancer, OVCAR8-DXR ovarian cancer, and A549-DXR lung cancer cells. It also increases the activation of AKT in drug-resistant cells, causing cellular resistance. Yun et al. (Yun et al., 2020) observed that Rp1 inhibited ActD-induced activation of the AKT/SIRT1 pathway and re-sensitized cells to ActD, reversing chemoresistance. Multidrug resistance to chemotherapeutic agents remains a major challenge in clinical cancer treatment. Feng et al. observed that Rg5 significantly reversed ABCB1-mediated multidrug resistance by increasing the intracellular accumulation of ABCB1 substrates without altering ABCB1 protein expression. The combination of Rg5 and docetaxel (TXT) enhanced therapeutic efficacy. They significantly inhibited the growth of drug-resistant tumors by inhibiting the AKT/Nrf2 pathway and significantly inhibited the growth of drug-resistant tumors, confirming that Rg5 combined with TXT treatment was superior to monotherapy without increased toxicity (Feng et al., 2020).

Gemcitabine (GEM) is one of the first-line drugs for the treatment of pancreatic cancer, however, its treatment effect is not long-lasting due to prolonged drug resistance. The combination of PNN and GEM inhibits Ki-67 and Bcl-2 protein expression in pancreatic cancer PANC-1 cells, decreases tumor cell stemness, inhibits their proliferation, and promotes their apoptosis (Yang L et al., 2019). β-sitosterol combined with GEM exhibits synergistic anti-pancreatic cancer activity by regulating apoptosis and inhibiting epithelial-mesenchymal transition through inhibition of Akt/GSK-3β signaling (Cao et al., 2020).

β-sitosterol further increased the limitation of cisplatin-and PTX-induced growth of human ovarian cancer cells, exhibiting synergistic anticancer effects (Bae et al., 2021). β-sitosterol reverses oxaliplatin resistance in drug-resistant colorectal cancer cells by inhibiting the p53-MDM2 interaction in colorectal cancer by activating p53, leading to increased p53 translocation to the nucleus and the NF-κB pathway, which in turn inhibits the breast cancer resistance protein (BCRP) (Wang Z et al., 2020).

Cinnamic acid, either as a single agent or in combination with cisplatin, significantly reduced tumor growth and volume and increased tumor growth inhibition. Mechanistically, cinnamic acid induced apoptosis by increasing the Bax/Bcl-2 ratio and caspase-3 expression (14.3 and 11.6-fold increases, respectively). Also, cinnamic acid combined with cisplatin decreased oxidative stress markers, including lipid peroxidation and nitric oxide levels and increased levels of reduced glutathione levels (Almeer et al., 2019).

β-elemene enhances antitumor activity and reverses chemoresistance to several chemotherapeutic agents. For example, the combination of β-elemene with 5-FU enhances the chemotherapeutic effect and reverses the chemoresistance of 5-FU in triple-negative breast cancer (Su et al., 2020) and p53-deficient colorectal cancer (Zhang S et al., 2020). Li et al. observed that β-elemene in combination with oxaliplatin, enhanced HCC sensitivity to oxaliplatin by blocking oxaliplatin-induced degradation of copper transporter protein 1 (Li X et al., 2016). In addition, β-elemene can be used in combination with erlotinib (Wang J et al., 2021), PTX (Xiaomeng et al., 2020), cetuximab (Chen et al., 2020), and gefitinib (Cheng et al., 2018) to enhance therapeutic effects, reduce drug resistance, and inhibit malignant tumor progression. Srijiwangsa et al. observed that β-eucalyptol enhanced the chemosensitivity of CCA cells to 5-FU and adriamycin (Srijiwangsa et al., 2018).

Radiotherapy is also one of the basic tools of oncology treatment; however, tumor radiation resistance remains a major treatment obstacle. GRg3 enhances the radiosensitivity of esophageal (Li et al., 2021b) and colorectal cancers (Liu T et al., 2018) by downregulating the VEGF, thereby reducing tumor size, inhibiting tumor growth, and prolonging survival. Nrf2, a key transcription factor that regulates resistance to oxidative stress, plays an important role in the induction of antioxidant responses in the body. Under physiological conditions, Nrf2 maintains cellular redox homeostasis and exerts anti-inflammatory and anticancer activities, thereby supporting cell survival. However, over-activation of Nrf2 also confers multiple advantages to cancer cells, such as protection from apoptosis and senescence, promotion of cell growth, and mediation of resistance to chemotherapy and radiotherapy (Su et al., 2019). Ashrafizadeh et al. observed that ginsenosides could improve the antioxidant defense system by inhibiting the Nrf2 signaling pathway and enhancing the activity of CAT, SOD, GSH, and GP, thus reducing malondialdehyde and lipid peroxidation levels, alleviating cellular drug resistance, and exerting antitumor effects (Ashrafizadeh et al., 2021). GPS also promote efficacy and reduce adverse effects (Zhu et al., 2021a). GPS treatment significantly induces OS cell death, and combination therapy with radiotherapy could improve the therapeutic effect (Zhang E et al., 2017). Bai et al. showed that β-elemene enhanced the radiosensitivity of gastric cancer cells SGC7901, MKN45, MKN28, N87, and AGS human gastric cancer cell lines by inhibiting Pak1 activation (Bai et al., 2021).

GS also plays a significant role in immunotherapy (Li Y et al., 2022) and targeted therapy (Jiang et al., 2017; Liu J et al., 2022). Jiang et al. observed that GRg3 enhances the anti-proliferative activity of erlotinib in pancreatic cancer cell lines by downregulating the EGFR/PI3K/Akt signaling pathway (Jiang et al., 2017).

## 4 Reduce the complications and improve the efficacy

Cancer cachexia is a serious disease that causes death in patients with advanced cancer, and approximately 50–80% of cancer patients suffer from cancer cachexia. Ginseng extracts reportedly have significant anticancer and immune-enhancing effects. GRb1 can ameliorate the symptoms of cancer cachexia by reducing TNF-α and IL-6 cytokine levels caused by inflammation in cancer cachectic mice (Lu et al., 2020).

The neuroprotective effects of β-sitosterol are associated with reduced levels of oxidative stress. Their antioxidant effects are attributed to the neutralization of free radicals by providing electrons directly from their hydroxyl or carboxyl groups (Zwolak, 2020). The cinnamic acid derivatives, caffeic acid and ferulic acid, improved the effectiveness of Transcatheter arterial embolization in the treatment of HCC by blocking lactate efflux from N1S1 tumor cells (significantly reduced by 90%) (Wilkins et al., 2017). Pancreatic cancer is one of the most lethal cancer types. Patients with advanced pancreatic cancer usually develop peritoneal effusion, which severely affects their quality of life. Zhu et al. observed that β-elemene could target and block the HIF1A/VEGFA pathway, thereby inhibiting the production of peritoneal effusion in pancreatic cancer (Zhu et al., 2019).

## 5 Clinical research

Transcatheter arterial chemoembolization (TACE) is the standard of care for HCC, however, its role is limited by multiple complications. Ginsenosides, including GRg3, GRh2, and GC-K, have been used clinically as adjuvants for TACE in the treatment of HCC. They also play an important role in enhancing the efficacy and reducing the adverse effects of HCC. A study evaluating the efficacy and safety of TACE combined with ginsenosides for the treatment of HCC included a total of 1, 308 patients with HCC from 18 randomized controlled trials. This study demonstrated that compared with TACE, the combination of ginsenosides significantly improved objective remission rates, disease control, quality of life, and 1–2 years OS and reduced the risk of adverse events (e.g., nausea, vomiting, fever, pain, hyperbilirubinemia, anorexia, fatigue, leukopenia, thrombocytopenia, and myelosuppression), with Rg3 being the preferred choice for the combination (Zhu et al., 2021b). A case-control study examining the relationship between dietary phytosterol intake and colorectal cancer risk in a Chinese population from July 2010 to June 2016 showed that the intake of total phytosterols and β-sitosterol was negatively associated with colorectal cancer risk in a Chinese population (Huang J et al., 2017). In another randomized double-blind trial, colorectal cancer patients treated with mFOLFOX-6 (n = 219) were included to assess the effect of ginseng on CRF. The results showed that compared to placebo, ginseng resulted in significant CRF over a 16-week period, particularly “Fatigue right now,” “Mood,” “Relations with others,” “Walking ability,” and “Enjoyment of life”. Thus, ginseng can be safely used in combination with chemotherapy in patients with colorectal cancer, reducing CRF compared with placebo (Kim et al., 2020).

## 6 New technologies/new drug delivery systems

Extracting highly active ingredients from ginseng for the prevention and treatment of diseases is important for the development of modern drugs. However, due to the different nature of the active ingredients of ginseng, the problem of its stability, solubility, irritation, and bioavailability must be solved to expand its clinical application. Moreover, the use of advanced drug delivery technology, carrier technology, and nanotechnology can effectively address the key problems in drug delivery.

The clinical treatment of gastric cancer is hampered by the development of anticancer drug resistance, unfavorable pharmacokinetics, off-target toxicity, and inadequate intratumoral accumulation of current chemotherapeutic treatments. The combination of GS and PTX synergistically inhibits of human gastric cancer cell proliferation. Hong et al. (Hong et al., 2019) established new ginsenoside-based liposomes for tumor-targeting therapies. GS acts as a chemotherapeutic adjuvant in combination with PTX and as a functional membrane material that promotes blood circulation time and active targeting ability to successfully deliver and internalize the drug into GC cells, significantly inhibiting the malignant progression of gastric cancer BGC-823 cells.

HCC is a highly vascularized, inflammatory, and abnormally proliferating tumor. Monotherapy is often not effective and comprehensive enough to inhibit HCC progression. Ren et al. (Ren et al., 2020) developed a novel nanomedicine, GRg3 nanoparticle adduct (NpRg3), by coupling Fe@Fe_3_O_4_ nanoparticles with ginsenoside Rg3. NpRg3 significantly inhibits HCC development and reshapes the network associated with an imbalance between gut microbiota and metabolism during HCC treatment, inhibiting HCC development and pulmonary metastasis. He et al. (He et al., 2021) selected GRg3, Ganoderma lucidum polysaccharide, and oridonin as combination therapies and constructed a new drug self-microemulsifying drug delivery system (RGO-SMEDDS). RGO-SMEDDS restores immune function by inhibiting the production of immunosuppressive cytokines and M2-polarized macrophages, reduces angiogenesis by downregulating the VEGF and its receptor, and retards proliferation by inhibiting the EGFR/AKT/GSK3 signaling pathway.

CRC is a major malignancy characterized by a high metastasis rate; however, conventional chemotherapy has many limitations (e.g., side effects on normal organs, short circulation time, and unsatisfactory tumor suppression effect) that affect its therapeutic effect. Therefore, it is necessary to develop approaches to overcome these challenges. Immune checkpoint blockade therapy is an emerging treatment in the field of cancer immunotherapy; however, the efficacy of immune checkpoint inhibitors in nodal CRC remains low due to the immunosuppressive nature of the TME. Emerging evidence suggests that certain chemotherapeutic agents induce immunogenic cell death (ICD), thus showing great potential to reshape the immunosuppressive properties of the TME. GRg3 plays a potential role as an ICD inducer in CRC cells. Quercetin (QTN) induces ROS production, which synergistically enhances the ICD effect of GRg3. To improve the *in vivo* delivery barriers associated with chemotherapeutic drugs, Sun et al. applied GRg3 combined with QTN to develop cyclodextrin nanoparticles (NP) and synthesized nanoformulations (CD-PEG-FA.Rg3. QTN), which significantly prolonged blood circulation, enhanced tumor targeting, and led to immunosuppressive TME conversion in an *in situ* CRC mouse model. Combination with CD-PEG-FA.Rg3. QTN with Anti-PD-L1 significantly prolonged the survival of animals (Sun et al., 2022). Qiu et al. prepared 20(S)-GRg3 (mPEG-b-P (Glu-co-Phe)) nanoparticles (Rg3-NPs). The mPEG-b-P (Glu-co-Phe)-based drug delivery system allows the rapid release of GRg3 from nanoparticles to target cancer cells, reduces the expression of nuclear antigens in proliferating cells, significantly inhibits CRC proliferation, and leads to CRC apoptosis by increasing the expression of caspase-3 (Qiu et al., 2019).

As advanced nanotechnology with special properties and structure, carbon nanotubes have shown practical drug delivery properties. Luo et al. (Luo et al., 2021) demonstrated that GRg3 containing carbon nanotubes (Rg3-CNT) could further enhance the antitumor effects of Rg3 by inhibiting proliferation and promoting apoptosis. Lahiani et al. (Lahiani et al., 2017) demonstrated that combined treatment with Rg1-CNT and Rb1-CNT reduced cell viability by 62% and enhanced the antiproliferative properties of drug-resistant pancreatic cancer cells (PANC-1) by 61%. Transient receptor potential (TRP) ion channels are a group of channel proteins widely distributed in the peripheral and central nervous systems. Abnormal activation of TRP channels can cause various diseases, including neurodegenerative diseases, cancer, skeletal dysplasia, and renal dysfunction. Li et al. demonstrated that G-Rd could inhibit gastric AGS cell proliferation by suppressing TRPM7-like currents in the TRPM7 channels (Li et al., 2022b).

Silver nanoparticles (AgNPs) are commonly used in healthcare systems as antibacterial, anti-viral, and anticancer agents due to their unique physicochemical and biological properties. β-sitosterol-mediated AgNPs induce concentration-dependent cytotoxicity and induces p53 expression in HT-29 cells (Shathviha et al., 2021). In addition, β-sitosterol-assisted silver nanoparticles upregulate the expression of proapoptotic markers, activating Nrf2 and oxidative stress and triggering mitochondrial apoptosis in HepG2 cells (Raj et al., 2020). However, β-sitosterol has poor water solubility and bioavailability and a short elimination half-life, which are a great limitation for its therapeutic application. To overcome these two drawbacks, β-sitosterol-loaded niosomes were prepared—the film hydration method and process parameters were optimized using a three-factor Box-Behnken design, and further surface modification with polyethylene glycol (PEG) was performed. *In vitro* and *in vivo* experiments showed that BSMF had improved therapeutic properties and significant cytotoxic potential for HCC with enhanced cellular uptake in HepG2 cells (Nisha et al., 2021). Block copolymers of poly (lactide-co-glycolic acid) (PLGA) and poly (ethylene glycol)-block-poly (lactic acid) (PEG-PLA) have been used to encapsulate β-sitosterol into nanoparticles designed to enhance its *in vitro* anticancer activity. Cell viability was inhibited by up to 80% in the concentration range of 6.64–53.08 μg/ml compared to untreated cells. In conclusion, the encapsulation of β-sitosterol in PLGA nanoparticles is a promising strategy to enhance its anticancer activity against breast cancer cells (Andima et al., 2018).

## 7 Prevention of precancerous lesions by the active ingredients of ginseng

Cancer occurrence and development is a long and gradual process. In its initial stage, the human body is in a deadly struggle with cancer cells, and many pre-cancerous “signs” will appear, indicating a benign diseases of the human tissues or organs. These “signs” may be some benign diseases of human tissues or organs. However, they often have the potential to become cancerous; if left untreated for a long time, a significant proportion of them will become cancerous. We refer to these diseases as “precancerous lesions”, and they are a group of lesions with special characteristics. These are neither malignant tumors nor malignant tumor states. However, under the continuous influence of multiple carcinogenic factors, precancerous lesions will have the tendency and possibility to develop further into malignant tumors. Common clinical precancerous lesions of the GI tract include: chronic atrophic gastritis and gastric ulcer (GU), chronic hepatitis and cirrhosis, colon and rectal polyps, and epithelial hyperplasia. (Wang Y et al., 2021).

Recently, many studies have shown that ginseng and its active ingredients have significant inhibitory effects on precancerous lesions. Zhang et al. observed that 20(S)-GRg3 significantly reduced UI scores and UI ratios in three GU models (alcohol GU, pylorus-ligated GU, and acetic acid GU models) and showed anti-ulcer effects and gastric mucosal protection by decreasing ET-1 and NOS2 levels and increasing NO, superoxide dismutase, EGF, and epidermal growth factor receptor levels (Zhang J et al., 2019). In addition, GRg3 inhibits the abnormal activation of Glut1 and Glut4 in AGS and HGC-27 human gastric cancer cells, alleviating microvascular abnormalities and thus inhibiting gastric precancerous lesion angiogenesis (Xu et al., 2021). β-linked protein upregulation and nuclear translocation are significantly associated with advanced gastric precancerous lesions (GPL). Zeng et al. (Zeng et al., 2021) observed that GRb1 reduced the protein expression and nuclear translocation of β-catenin, interfered with β-catenin/TCF4 interactions, and prevented the development and progression of GPL.

Non-alcoholic fatty liver disease (NAFLD) is a metabolic liver disease with complex etiology and is considered one of the major causes of HCC development. Zhang et al. (Zhang J et al., 2022) observed that ginsenoside C-K reduced lipid deposition in HepG2 cells, attenuated lipid accumulation in serum and liver tissues, ameliorated liver inflammation and injury, regulated the expression of factors related to lipid synthesis and metabolism, activated the phosphorylation of LKB1 and AMPK, and intervened in the progression of NAFLD. In addition, Li et al. (Li et al., 2022a) observed that GRg1 significantly downregulated the expression level of sphingosine-1-phosphate lyase 1 (SGPL1) and increased the expression levels of p-Akt and p-ERK1/2 in steatosis HHL 5 cells, exerting an anti-apoptotic effect on nonalcoholic fatty liver cells. Previous reports have shown that GRk3 has excellent efficacy in reducing the intestinal inflammatory response and protecting the liver. Qu et al. observed that the application of GRk3 inhibited liver injury, fibrosis, and cirrhosis and had strong antitumor effects in a mouse model of dimethylnitrosamine and CCL4-induced HCC. In addition, GRk3 can reduce the expression of inflammatory cytokines, improve intestinal flora, inhibit the LPS-TLR4 signaling pathway, and play a key role in HCC prevention (Qu Y et al., 2021).

Rectal polyps are major risk factors for rectal cancer. Significantly higher levels of IL-4, MIP-1β, FasL, and TGF-β1 were detected in rectal polyps. Zhang et al. (Xie et al., 2019) observed that regular administration of Rg3 may prevent rectal polyps by lowering the serum levels of selected cytokines (including IL-4, MIP-1β, FasL, and TGF-β1). Huang et al. (Huang G et al., 2017) observed that Rb3/Rd prevented precancerous lesions by downregulating oncogenic signaling molecules (iNOS, STAT3/pSTAT3, and Src/pSrc) to reduce the size and number of polyps. Nodules are also an important factors in the development of neoplastic lesions. Treatment with 20(R)-Rg3 also reduces the number and size of nodules in the liver, lung and kidney tissues and has a preventive effect against precancerous lesions (Phi et al., 2019).

Inflammatory colitis, caused by obesity, is also a major cause of colorectal cancer. Chen et al. observed that GRk3 repaired intestinal barrier dysfunction by increasing the expression of tight junction proteins (occludin-1, claudin, and occludin) and reducing colonic inflammatory cytokine levels, oxidative stress, and macrophage infiltration. In addition, GRk3 effectively improved dysregulated intestinal flora metabolism by inhibiting the TLR4/NF-κB signaling pathway, significantly reducing the thick-walled flora/mimic flora ratio and inhibiting the inflammatory cascade response (Chen X et al., 2021). Chen et al. observed that ginsenoside GRh2 alleviated ulcerative colitis by downregulating the expression of pro-inflammatory cytokines TNF-α, IL-6, and IL-1β by regulating the STAT3/miR-214 signaling pathway (Chen C et al., 2021).

Tovey observed that stigmasterol activates the activity of membrane-bound enzymes and fluidity of cell membranes, which in turn exerts a protective effect on the gastric mucosa (Tovey, 2015). Pandith et al. observed that stigmasterol has anti-inflammatory and antitumor effects and can significantly reduce the expression of COX-2 and inducible nitric oxide synthase protein, exerting anti-GI effects (Pandith et al., 2013). Feng et al. (Feng et al., 2018) also demonstrated that soy sterols significantly inhibited dextran sodium sulfate-induced colonic shortening in C57BL/6J male mice, reduced fecal hemoglobin content, decreased colitis pathology scores, and significantly reduced the mRNA expression of colonic tissue inflammatory factors IL-1, IL-6, MCP-1, and COX-2. In addition, stigmasterol inhibits lipopolysaccharide-induced innate immune responses in mice, suppresses the proliferation of inflammatory cells in the blood of model mice, and effectively controls colonic tissue damage (Antwi et al., 2017).

Eugenol and cinnamic acid significantly inhibit HCl/ethanol-induced gastric damage, increase mucus content, have antioxidant activity, and exhibit protective effects against gastric damage *in vivo* (Jung et al., 2011). Cinnamic acid derivatives are potent antiviral compounds against the hepatitis C virus (HCV). The cinnamic acid derivative AG490 inhibits HCV replication and JAK2 activity. Cinnamic acid compound 6 effectively inhibits HCV replication and induces ROS production (Amano et al., 2017).

## Discussion

Cancer is a complex systemic chronic disease that threatens human health and quality of life. Ginseng, a traditional natural product, has unparalleled advantages in the treatment of digestive tumors because of its wide range of pharmacological activities and application pathways. This review summarizes the use of ginseng and its active ingredients for the treatment of GI tumors and focuses on the antitumor mechanism of action of ginseng, the combined application of ginseng and modern medicine, clinical studies, novel drug delivery techniques and the prevention of precancerous lesions, thereby inhibiting tumorigenesis and malignant progression. (Table 1).

**TABLE 1:** Tumor types and characteristics of various active components of ginseng.

Type	Composition	Tumor type	Synergistic reaction	Features
Ginsenosides and Their Metabolites	Rb1, Rb2, Rb3、Rc, Rd, Rg3, Rg5, Rh2, Rs11, Rk1, F2, CK, Re, Re7, Rg1, Rg18, Rh1, Rh4, Rp1, Rf, F1, PPD, 25-OH-PPD, 25-OCH_3_-PPD	Esophageal, Stomach, Liver, Colon, Pancreas, and Gallbladder	Cisplatin, Docetaxel, Doxorubicin, Gemcitabine, Oxaliplatin, Sorafenib and 5-FU	①Wide range of anti-tumor effects
Ginseng Polysaccharides	GFP1, PGP2a、PGPW1, Ginsan、WGPA-1-HG, 2-HG, 3-HG, 4-HG, WGPA-3-RG, 4-RG	Stomach, Liver, Colon	-	②Anti-tumor components are complex and time- and dose-dependent
Ginseng Polyacetylenes	PND, PNT, PNN	Liver, Pancreas, and Colon	Gemcitabine	③Anti-tumor effects are exerted through multiple pathways
Sterols	β-Sitosterl, Stigmasterol	Stomach, Liver, Colorectal, and Gallbladder	Cisplatin, Gemcitabine, Oxaliplatin and Paclitaxel	④Synergistic effects can be produced in combination with multiple anti-tumor therapies to improve efficacy and reduce toxic side effects
Volatile Oils	β-Panasinsene	Stomach, Liver, Colorectal, Pancreas, and Bile Duct	Cetuximab, Doxorubicin, Erlotinib, Gefitinib, Oxaliplatin, Paclitaxel and 5-FU	⑤Combined with novel drug delivery systems
	α-Gurjunene, Germacrene			
	β-Gurjunene			
	β-Elemene			
	β-Caryophyllene			
	β-Neoclovene			
Organic Acids	Citric Acid, Cinnamic Acid, Fumaric Acid, Maleic Acid, Salicylic Acid	Liver, Colorecta	Cisplatin	⑥Prevention of precancerous lesions

**FIGURE 2:**
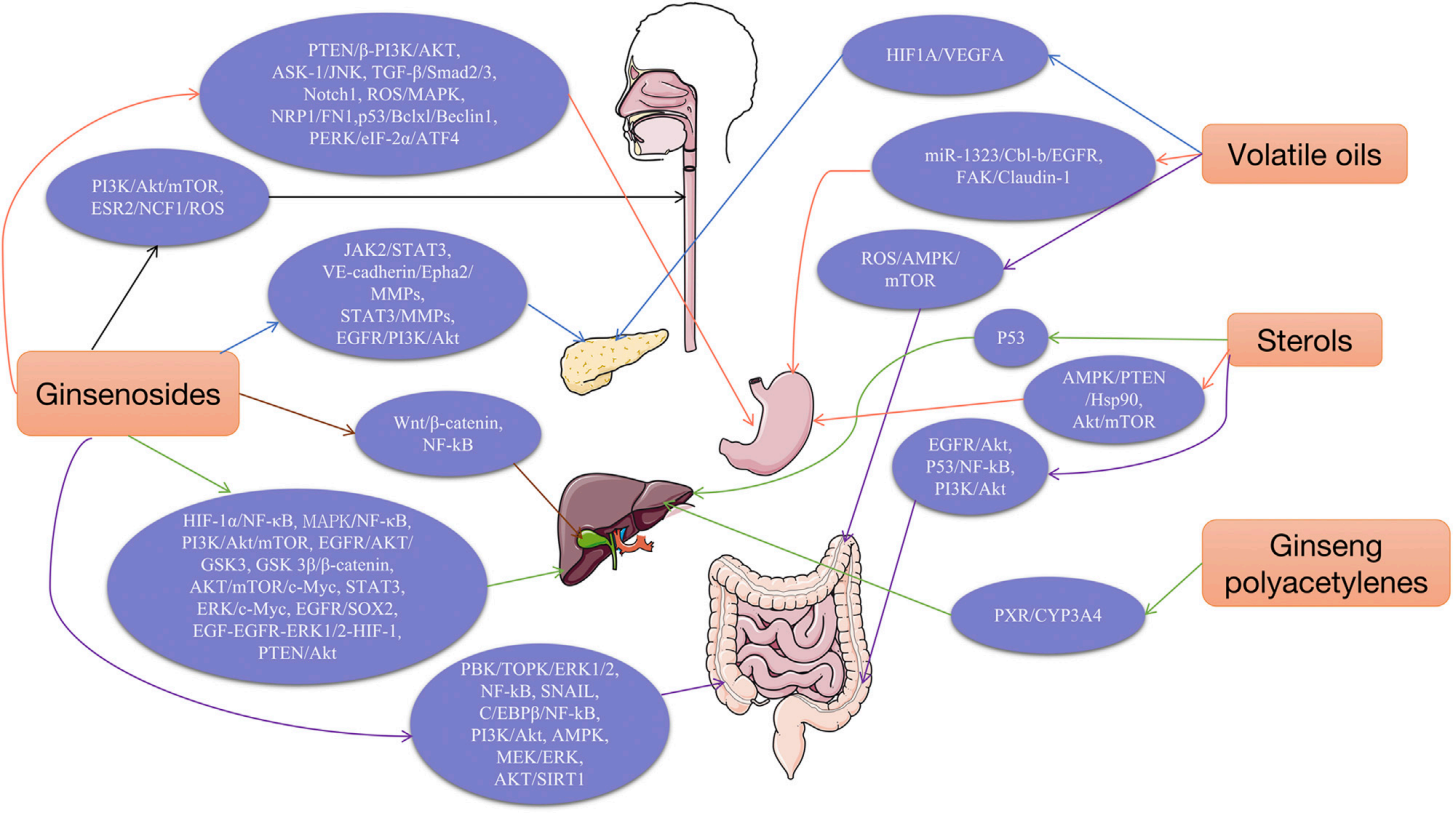
Molecular mechanism/pathway of the anti-tumor effects of ginseng.

As shown in Table 1, the active ingredients of ginseng are very complex, and its main active ingredients, such as ginsenosides, can fight most common tumors. Other ginseng active ingredients may also have potential antitumor activity, although they account for a small percentage, requiring further research. In conclusion, research on the antitumor mechanism of the active ingredients of ginseng, especially monomeric ginsenosides, has made great progress, and its molecular mechanism mainly involves the regulation and expression of many related genes, proteins, proteases, immune cells, cytokines, and related signaling pathways, as shown in Figure 2.

As shown in Figure 2, active ingredients of ginseng can regulate many related signaling pathways, including PI3K/Akt/mTOR, MAPKs, JAK/STAT, Wnt/β-catenin, AMPK, MEK, EGFR, NF-κB, and TGF-β and so on. Ginsenosides and other components can directly or indirectly regulate these pathways, thus acting on signaling targets to exert antitumor effects.

Several experimental data have proven that ginseng has definite antitumor effects, and its pharmacological substance basis and molecular mechanism of action have been clarified; however, there are also some defects and shortcomings. Moreover, most of the current research results are based on *in vitro* cellular experiments and relatively little *in vivo* animal experimental and clinical trial data; therefore, we should focus on the combination of basic experiments and clinical trials. Recently, the use of advanced drug delivery technologies, carrier technologies, and nanotechnology in modern pharmacology has effectively improved the stability, solubility, bioavailability, and other key issues in drug delivery; however, their applications are limited to a few ginsenosides, and research on novel drug delivery technologies for other active ingredients of ginseng is still relatively rare. Although the combined treatment method of ginseng and modern medicine has begun to be recognized, there is still a huge resistance and difficulties in its application.

Therefore, in future in-depth research and development, it is necessary to improve the following aspects: 1) in-depth exploration of the molecular mechanism of action of the antitumor effects of the active ingredients of ginseng using modern molecular biology techniques and their validation using *in vivo* experiments; 2) to study the connection between the relevant signal transduction pathways involved in the antitumor effects of ginseng; 3) comparative studies on the antitumor activity of various active ingredients to select the best combination for a particular tumor; 4) to conduct in-depth research on GPS and ginseng polyacetylenes using advanced drug delivery technology, carrier technology and nanotechnology in modern pharmacology. Ginseng is rich in a variety of antitumor active ingredients and has a promising future as an anticancer adjuvant drug candidate. Moreover, ginseng and its components contribute to the therapeutic efficacy of modern medicine and can significantly improve the quality of life while minimizing the adverse effects of modern medicine. Currently, the monomeric ginsenoside Rg3 has been developed as a new class I drug for adjuvant treatment of various cancers. Hence, we expect that more active ginseng ingredients will be available in the future to provide safe and effective natural drugs and agents for the clinical treatment of various tumors.

## References

[B1] Ahmed E. A. Rajendran P. Scherthan H. (2022). The microRNA-202 as a diagnostic biomarker and a potential tumor suppressor. Int. J. Mol. Sci. 23 (11), 5870. 10.3390/ijms23115870 35682549 PMC9180238

[B2] Almeer R. S. Aref A. M. Hussein R. A. Othman M. S. Abdel M. A. (2019). Antitumor potential of berberine and cinnamic acid against solid ehrlich carcinoma in mice. Anticancer. Agents Med. Chem. 19 (3), 356–364. 10.2174/1871520618666181116162441 30451117

[B3] Amano R. Yamashita A. Kasai H. Hori T. Miyasato S. Saito S. (2017). Cinnamic acid derivatives inhibit hepatitis C virus replication via the induction of oxidative stress. Antivir. Res. 145, 123–130. 10.1016/j.antiviral.2017.07.018 28780423

[B4] Amin E. Jaiswal M. Derewenda U. Reis K. Nouri K. Koessmeier K. T. (2016). Deciphering the molecular and functional basis of rhogap family proteins: A systematic approach toward selective inactivation of Rho family proteins. J. Biol. Chem. 291 (39), 20353–20371. 10.1074/jbc.M116.736967 27481945 PMC5034035

[B5] Anantharaju P. G. Reddy D. B. Padukudru M. A. Chitturi C. Vimalambike M. G. Madhunapantula S. V. (2017). Induction of colon and cervical cancer cell death by cinnamic acid derivatives is mediated through the inhibition of Histone Deacetylases (HDAC). PLoS One 12 (11), e0186208. 10.1371/journal.pone.0186208 29190639 PMC5708809

[B6] Andima M. Costabile G. Isert L. Ndakala A. J. Derese S. Merkel O. M. (2018). Evaluation of beta-sitosterol loaded PLGA and PEG-PLA nanoparticles for effective treatment of breast cancer: Preparation, physicochemical characterization, and antitumor activity. Pharmaceutics 10 (4), E232. 10.3390/pharmaceutics10040232 30445705 PMC6321471

[B7] Antwi A. O. Obiri D. D. Osafo N. Essel L. B. Forkuo A. D. Atobiga C. (2018). Stigmasterol alleviates cutaneous allergic responses in rodents. Biomed. Res. Int. 2018, 3984068. 10.1155/2018/3984068 30140696 PMC6081592

[B8] Antwi A. O. Obiri D. D. Osafo N. Forkuo A. D. Essel L. B. (2017). Stigmasterol inhibits lipopolysaccharide-induced innate immune responses in murine models. Int. Immunopharmacol. 53, 105–113. 10.1016/j.intimp.2017.10.018 29078089

[B9] Ashrafizadeh M. Ahmadi Z. Yaribeygi H. Sathyapalan T. Jamialahmadi T. Sahebkar A. (2021). The effects of ginsenosides on the Nrf2 signaling pathway. Adv. Exp. Med. Biol. 1328, 307–322. 10.1007/978-3-030-73234-9_20 34981486

[B10] Aziz F. Wang X. Liu J. Yan Q. (2016). Ginsenoside Rg3 induces FUT4-mediated apoptosis in *H. pylori* CagA-treated gastric cancer cells by regulating SP1 and HSF1 expressions. Toxicol. Vitro. 31, 158–166. 10.1016/j.tiv.2015.09.025 26427350

[B11] Bae H. Park S. Ham J. Song J. Hong T. Choi J. H. (2021). ER-mitochondria calcium flux by beta-sitosterol promotes cell death in ovarian cancer. Antioxidants (Basel) 10 (10), 1583. 10.3390/antiox10101583 34679718 PMC8533280

[B12] Bae H. Song G. Lim W. (2020). Stigmasterol causes ovarian cancer cell apoptosis by inducing endoplasmic reticulum and mitochondrial dysfunction. Pharmaceutics 12 (6), E488. 10.3390/pharmaceutics12060488 32481565 PMC7356731

[B13] Bai Z. Yao C. Zhu J. Xie Y. Ye X. Y. Bai R. (2021). Anti-tumor drug discovery based on natural product beta-elemene: Anti-tumor mechanisms and structural modification. Molecules 26 (6), 1499. 10.3390/molecules26061499 33801899 PMC7998186

[B14] Banyard J. Bielenberg D. R. (2015). The role of EMT and MET in cancer dissemination. Connect. Tissue Res. 56 (5), 403–413. 10.3109/03008207.2015.1060970 26291767 PMC4780319

[B15] Bin S. M. Ameen S. S. (2015). Beta-sitosterol: A promising but orphan nutraceutical to fight against cancer. Nutr. Cancer. 67 (8), 1214–1220. 10.1080/01635581.2015.1087042 26473555

[B16] Bomfim D. S. Ferraz R. P. Carvalho N. C. Soares M. B. Pinheiro M. L. Costa E. V. (2013). Eudesmol isomers induce caspase-mediated apoptosis in human hepatocellular carcinoma HepG2 cells. Basic Clin. Pharmacol. Toxicol. 113 (5), 300–306. 10.1111/bcpt.12097 23786320

[B17] Boubaker J. Ben T. I. Sassi A. Bzouich-Mokded I. Ghoul M. S. Sioud F. (2018). Antitumoral potency by immunomodulation of chloroform extract from leaves of nitraria retusa, Tunisian medicinal plant, via its major compounds beta-sitosterol and palmitic acid in BALB/c mice bearing induced tumor. Nutr. Cancer. 70 (4), 650–662. 10.1080/01635581.2018.1460683 29697283

[B18] Cai J. Jing J. Lu Y. Du R. (2007). Research progress of natural polyacetylene alcohol. Tianjin: Chinese Traditional and Herbal Drugs, 620–623.04.

[B19] Cao Y. Ye Q. Zhuang M. Xie S. Zhong R. Cui J. (2017). Ginsenoside Rg3 inhibits angiogenesis in a rat model of endometriosis through the VEGFR-2-mediated PI3K/Akt/mTOR signaling pathway. PLoS One 12 (11), e0186520. 10.1371/journal.pone.0186520 29140979 PMC5687597

[B20] Cao Z. Q. Wang X. X. Lu L. Xu J. W. Li X. B. Zhang G. R. (2020). Corrigendum: β-Sitosterol and gemcitabine exhibit synergistic anti-pancreatic cancer activity by modulating apoptosis and inhibiting epithelial-mesenchymal transition by deactivating akt/GSK-3β signaling. Front. Pharmacol. 11, 565535. 10.3389/fphar.2020.565535 33658921 PMC7919186

[B21] Chen C C. Lv Q. Li Y. Jin Y. H. (2021). The anti-tumor effect and underlying apoptotic mechanism of ginsenoside Rk1 and Rg5 in human liver cancer cells. Molecules 26 (13), 3926. 10.3390/molecules26133926 34199025 PMC8271777

[B22] Chen H. Yang H. Deng J. Fan D. (2021). Ginsenoside Rk3 ameliorates obesity-induced colitis by regulating of intestinal flora and the TLR4/NF-κB signaling pathway in C57bl/6 mice. J. Agric. Food Chem. 69 (10), 3082–3093. 10.1021/acs.jafc.0c07805 33621094

[B23] Chen P. Li X. Zhang R. Liu S. Xiang Y. Zhang M. (2020). Combinative treatment of beta-elemene and cetuximab is sensitive to KRAS mutant colorectal cancer cells by inducing ferroptosis and inhibiting epithelial-mesenchymal transformation. Theranostics 10 (11), 5107–5119. 10.7150/thno.44705 32308771 PMC7163451

[B24] Chen W. Chu S. Li H. Qiu Y. (2018). MicroRNA-146a-5p enhances ginsenoside Rh2-induced anti-proliferation and the apoptosis of the human liver cancer cell line HepG2. Oncol. Lett. 16 (4), 5367–5374. 10.3892/ol.2018.9235 30197683 PMC6126334

[B25] Chen X. Xu T. Lv X. Zhang J. Liu S. (2021). Ginsenoside Rh2 alleviates ulcerative colitis by regulating the STAT3/miR-214 signaling pathway. J. Ethnopharmacol. 274, 113997. 10.1016/j.jep.2021.113997 33705918

[B26] Cheng H. Ge X. Zhuo S. Gao Y. Zhu B. Zhang J. (2018). β-Elemene synergizes with gefitinib to inhibit stem-like phenotypes and progression of lung cancer via down-regulating EZH2. Front. Pharmacol. 9, 1413. 10.3389/fphar.2018.01413 30555330 PMC6284059

[B27] Cheng H. Li S. Fan Y. Gao X. Hao M. Wang J. (2011). Comparative studies of the antiproliferative effects of ginseng polysaccharides on HT-29 human colon cancer cells. Med. Oncol. 28 (1), 175–181. 10.1007/s12032-010-9449-8 20165990

[B28] Cheng L. Li H. Kong L. Yang J. Kang T. Zhang J. (2016). Ginseng proteins synergize with H_2O_2 to induce oxidative damage in SH-SY5Y cells. Chin. J. Hosp. Pharm. | Chin J Hosp Pharm. 36 (09), 707–710.

[B29] Cheng Z. Xing D. (2019). Retracted: Ginsenoside Rg3 inhibits growth and epithelial-mesenchymal transition of human oral squamous carcinoma cells by down-regulating miR-221. Eur. J. Pharmacol. 853, 353–363. 10.1016/j.ejphar.2019.03.040 30928631

[B30] Cong Z. Zhao Q. Yang B. Cong D. Zhou Y. Lei X. (2020). Ginsenoside Rh3 inhibits proliferation and induces apoptosis of colorectal cancer cells. Pharmacology 105 (5-6), 329–338. 10.1159/000503821 31671429

[B31] Conlon G. A. Murray G. I. (2019). Recent advances in understanding the roles of matrix metalloproteinases in tumour invasion and metastasis. J. Pathol. 247 (5), 629–640. 10.1002/path.5225 30582157

[B32] Cuypers A. Truong A. K. Becker L. M. Saavedra-Garcia P. Carmeliet P. (2022). Tumor vessel co-option: The past & the future. Front. Oncol. 12, 965277. 10.3389/fonc.2022.965277 36119528 PMC9472251

[B33] de Oliveira J. R. Christiane A. A. Da S. A. J. Grougnet R. Thiery V. Picot L. (2018). Sensitization of tumor cells to chemotherapy by natural products: A systematic review of preclinical data and molecular mechanisms. Fitoterapia 129, 383–400. 10.1016/j.fitote.2018.02.025 29476786

[B34] De Wang X. Li T. Li Y. Yuan W. H. Zhao Y. Q. (2020). 2-Pyrazine-PPD, a novel dammarane derivative, showed anticancer activity by reactive oxygen species-mediate apoptosis and endoplasmic reticulum stress in gastric cancer cells. Eur. J. Pharmacol. 881, 173211. 10.1016/j.ejphar.2020.173211 32464194

[B35] Deng M. Liu B. Song H. Yu R. Zou D. Chen Y. (2020). β-Elemene inhibits the metastasis of multidrug-resistant gastric cancer cells through miR-1323/Cbl-b/EGFR pathway. Phytomedicine 69, 153184. 10.1016/j.phymed.2020.153184 32199253

[B36] Deng M. Zhang Y. Liu B. Chen Y. Song H. Yu R. (2019). β-Elemene inhibits peritoneal metastasis of gastric cancer cells by modulating FAK/Claudin-1 signaling. Phytother. Res. 33 (9), 2448–2456. 10.1002/ptr.6436 31342604

[B37] Deng X. Zhao J. Qu L. Duan Z. Fu R. Zhu C. (2020). Ginsenoside Rh4 suppresses aerobic glycolysis and the expression of PD-L1 via targeting AKT in esophageal cancer. Biochem. Pharmacol. 178, 114038. 10.1016/j.bcp.2020.114038 32422139

[B38] Diener C. Keller A. Meese E. (2022). Emerging concepts of miRNA therapeutics: From cells to clinic. Trends Genet. 38 (6), 613–626. 10.1016/j.tig.2022.02.006 35303998

[B39] Ding P. Guo Y. Wang C. Chen J. Guo C. Liu H. (2022). A network pharmacology approach for uncovering the antitumor effects and potential mechanisms of the Sijunzi decoction for the treatment of gastric cancer. Evid. Based. Complement. Altern. Med. 2022, 9364313. 10.1155/2022/9364313 PMC9019414 35463069

[B40] Ditty M. J. Ezhilarasan D. (2021). β-sitosterol induces reactive oxygen species-mediated apoptosis in human hepatocellular carcinoma cell line. Avicenna J. Phytomed. 11 (6), 541–550. 10.22038/AJP.2021.17746 34804892 PMC8588954

[B41] Fan X. Fu H. Xie N. Guo H. Fu T. Shan Y. (2021). Inhibition of JAK2/STAT3 signaling pathway by panaxadiol limits the progression of pancreatic cancer. Aging (Albany NY) 13 (19), 22830–22842. 10.18632/aging.203575 34623971 PMC8544303

[B42] Feng L. S. Cheng J. B. Su W. Q. Li H. Z. Xiao T. Chen D. A. (2022). Cinnamic acid hybrids as anticancer agents: A mini-review. Arch. Pharm. Weinh. 355, e2200052. 10.1002/ardp.202200052 35419808

[B43] Feng S. L. Luo H. B. Cai L. Zhang J. Wang D. Chen Y. J. (2020). Ginsenoside Rg5 overcomes chemotherapeutic multidrug resistance mediated by ABCB1 transporter: *In vitro* and *in vivo* study. J. Ginseng Res. 44 (2), 247–257. 10.1016/j.jgr.2018.10.007 32148406 PMC7031741

[B44] Feng S. Ning K. Shao P. Ren G. Sun P. Luo Z. (2018). Therapeutic effect of β-sitosterol and stigmasterol on acute colitis in mice. J. Chin. Cereals Oils Assoc. 33 (12), 80–86+94.

[B45] Gangwar V. Garg A. Lomore K. Korla K. Bhat S. S. Rao R. P. (2021). Immunomodulatory effects of a concoction of natural bioactive compounds-mechanistic insights. Biomedicines 9 (11), 1522. 10.3390/biomedicines9111522 34829751 PMC8615223

[B46] Gebert L. Macrae I. J. (2019). Regulation of microRNA function in animals. Nat. Rev. Mol. Cell Biol. 20 (1), 21–37. 10.1038/s41580-018-0045-7 30108335 PMC6546304

[B47] Guo L. Song L. Wang Z. Zhao W. Mao W. Yin M. (2009). Panaxydol inhibits the proliferation and induces the differentiation of human hepatocarcinoma cell line HepG2. Chem. Biol. Interact. 181 (1), 138–143. 10.1016/j.cbi.2009.04.015 19450571

[B48] Guo W. Du K. Luo S. Hu D. (2022). Recent advances of autophagy in non-small cell lung cancer: From basic mechanisms to clinical application. Front. Oncol. 12, 861959. 10.3389/fonc.2022.861959 35600411 PMC9115384

[B49] Ham J. Jeong D. Park S. Kim H. W. Kim H. Kim S. J. (2019). Ginsenoside Rg3 and Korean Red Ginseng extract epigenetically regulate the tumor-related long noncoding RNAs RFX3-AS1 and STXBP5-AS1. J. Ginseng Res. 43 (4), 625–634. 10.1016/j.jgr.2019.02.004 31700260 PMC6823807

[B50] Hamilton P. T. Anholt B. R. Nelson B. H. (2022). Tumour immunotherapy: Lessons from predator-prey theory. Nat. Rev. Immunol. 1, 1. 10.1038/s41577-022-00719-y 35513493

[B51] Han B. Jiang Y. Zhang C. Zhou Y. Wang Y. (2012). Effect of 20(S)-ginsenoside Rg3 on the proliferation and apoptosis of colon cancer stem cells. Chin. J. Gerontology 32 (20), 4431–4433.

[B52] Han Q. Han L. Tie F. Wang Z. Ma C. Li J. (2020). (20S)-Protopanaxadiol ginsenosides induced cytotoxicity via blockade of autophagic flux in HGC-27 cells. Chem. Biodivers. 17 (7), e2000187. 10.1002/cbdv.202000187 32384197

[B53] Han S. Jeong A. J. Yang H. Bin K. K. Lee H. Yi E. H. (2016). Ginsenoside 20(S)-Rh2 exerts anti-cancer activity through targeting IL-6-induced JAK2/STAT3 pathway in human colorectal cancer cells. J. Ethnopharmacol. 194, 83–90. 10.1016/j.jep.2016.08.039 27566200

[B54] He F. Yan Q. Fan L. Liu Y. Cui J. Wang J. (2010). PBK/TOPK in the differential diagnosis of cholangiocarcinoma from hepatocellular carcinoma and its involvement in prognosis of human cholangiocarcinoma. Hum. Pathol. 41 (3), 415–424. 10.1016/j.humpath.2009.05.016 19954816

[B55] He S. Tian S. He X. Le X. Ning Y. Chen J. (2021). Multiple targeted self-emulsifying compound RGO reveals obvious anti-tumor potential in hepatocellular carcinoma. Mol. Ther. Oncolytics 22, 604–616. 10.1016/j.omto.2021.08.008 34589579 PMC8449031

[B56] Hong C. Wang D. Liang J. Guo Y. Zhu Y. Xia J. (2019). Novel ginsenoside-based multifunctional liposomal delivery system for combination therapy of gastric cancer. Theranostics 9 (15), 4437–4449. 10.7150/thno.34953 31285771 PMC6599661

[B57] Hong H. Baatar D. Hwang S. G. (2021). Anticancer activities of ginsenosides, the main active components of ginseng. Evid. Based. Complement. Altern. Med. 2021, 8858006. 10.1155/2021/8858006 PMC7875636 33623532

[B58] Hong S. Cai W. Huang Z. Wang Y. Mi X. Huang Y. (2020). Ginsenoside Rg3 enhances the anticancer effect of 5FU in colon cancer cells via the PI3K/AKT pathway. Oncol. Rep. 44 (4), 1333–1342. 10.3892/or.2020.7728 32945504 PMC7448421

[B59] Hu Q. (2018). Study of the regulation mechanism of CYP3A4 in HepG2 cells by ginsenoside triol based on nuclear receptor CAR. Nanchang: Nanchang University, 88.

[B60] Hu Q. Yao N. Wu J. Liu M. Liu F. Zhang H. (2019). Constitutive androstane receptor weakens the induction of panaxytriol on CYP3A4 by repressing the activation of pregnane X receptor. Biochem. Pharmacol. 159, 32–39. 10.1016/j.bcp.2018.11.009 30414935

[B61] Hu S. Zhu Y. Xia X. Xu X. Chen F. Miao X. (2019). Ginsenoside Rg3 prolongs survival of the orthotopic hepatocellular carcinoma model by inducing apoptosis and inhibiting angiogenesis. Anal. Cell. Pathol. 2019, 3815786. 10.1155/2019/3815786 PMC6732603 31534898

[B62] Huang G. Khan I. Li X. Chen L. Leong W. Ho L. T. (2017). Ginsenosides Rb3 and Rd reduce polyps formation while reinstate the dysbiotic gut microbiota and the intestinal microenvironment in Apc(Min/+) mice. Sci. Rep. 7 (1), 12552. 10.1038/s41598-017-12644-5 28970547 PMC5624945

[B63] Huang J. Xu M. Fang Y. J. Lu M. S. Pan Z. Z. Huang W. Q. (2017). Association between phytosterol intake and colorectal cancer risk: A case-control study. Br. J. Nutr. 117 (6), 839–850. 10.1017/S0007114517000617 28382872

[B64] Huang Y. Huang H. Han Z. Li W. Mai Z. Yuan R. (2019). Ginsenoside Rh2 inhibits angiogenesis in prostate cancer by targeting CNNM1. J. Nanosci. Nanotechnol. 19 (4), 1942–1950. 10.1166/jnn.2019.16404 30486934

[B65] Ishay-Ronen D. Diepenbruck M. Kalathur R. Sugiyama N. Tiede S. Ivanek R. (2019). Gain fat-lose metastasis: Converting invasive breast cancer cells into adipocytes inhibits cancer metastasis. Cancer Cell 35 (1), 17–32. e6. 10.1016/j.ccell.2018.12.002 30645973

[B66] Jang H. J. Han I. H. Kim Y. J. Yamabe N. Lee D. Hwang G. S. (2014). Anticarcinogenic effects of products of heat-processed ginsenoside Re, a major constituent of ginseng berry, on human gastric cancer cells. J. Agric. Food Chem. 62 (13), 2830–2836. 10.1021/jf5000776 24666263

[B67] Jiang H. Ma P. Duan Z. Liu Y. Shen S. Mi Y. (2022). Ginsenoside Rh4 suppresses metastasis of gastric cancer via SIX1-dependent TGF-β/smad2/3 signaling pathway. Nutrients 14 (8), 1564. 10.3390/nu14081564 35458126 PMC9032069

[B68] Jiang J. Yuan Z. Sun Y. Bu Y. Li W. Fei Z. (2017). Ginsenoside Rg3 enhances the anti-proliferative activity of erlotinib in pancreatic cancer cell lines by downregulation of EGFR/PI3K/Akt signaling pathway. Biomed. Pharmacother. 96, 619–625. 10.1016/j.biopha.2017.10.043 29035827

[B69] Jiang M. Zhu Y. Yu H. (2021). Ginsenoside 20(S)-Rg3 suppresses cell viability in esophageal squamous cell carcinoma via modulating miR-324-5p-targeted PSME3. Hum. Exp. Toxicol. 40 (11), 1974–1984. 10.1177/09603271211017311 34002647

[B70] Jung J. Lee J. H. Bae K. H. Jeong C. S. (2011). Anti-gastric actions of eugenol and cinnamic acid isolated from Cinnamomi Ramulus. Yakugaku Zasshi 131 (7), 1103–1110. 10.1248/yakushi.131.1103 21720141

[B71] Kang K. A. Kim H. S. Kim D. H. Hyun J. W. (2013). The role of a ginseng saponin metabolite as a DNA methyltransferase inhibitor in colorectal cancer cells. Int. J. Oncol. 43 (1), 228–236. 10.3892/ijo.2013.1931 23652987

[B72] Kangsamaksin T. Chaithongyot S. Wootthichairangsan C. Hanchaina R. Tangshewinsirikul C. Svasti J. (2017). Lupeol and stigmasterol suppress tumor angiogenesis and inhibit cholangiocarcinoma growth in mice via downregulation of tumor necrosis factor-α. PLoS One 12 (12), e0189628. 10.1371/journal.pone.0189628 29232409 PMC5726636

[B73] Kawk H. W. Nam G. H. Kim M. J. Kim S. Y. Kim Y. M. (2021). Scaphium affine ethanol extract induces anoikis by regulating the EGFR/Akt pathway in HCT116 colorectal cancer cells. Front. Oncol. 11, 621346. 10.3389/fonc.2021.621346 34094906 PMC8173041

[B74] Khan Z. Nath N. Rauf A. Emran T. B. Mitra S. Islam F. (2022). Multifunctional roles and pharmacological potential of beta-sitosterol: Emerging evidence toward clinical applications. Chem. Biol. Interact. 365, 110117. 10.1016/j.cbi.2022.110117 35995256

[B75] Kim H. S. Lim J. M. Kim J. Y. Kim Y. Park S. Sohn J. (2016). Panaxydol, a component of Panax ginseng, induces apoptosis in cancer cells through EGFR activation and ER stress and inhibits tumor growth in mouse models. Int. J. Cancer. 138 (6), 1432–1441. 10.1002/ijc.29879 26421996

[B76] Kim J. W. Han S. W. Cho J. Y. Chung I. J. Kim J. G. Lee K. H. (2020). Korean red ginseng for cancer-related fatigue in colorectal cancer patients with chemotherapy: A randomised phase III trial. Eur. J. Cancer. 130, 51–62. 10.1016/j.ejca.2020.02.018 32172198

[B77] Kim J. Y. Lee K. W. Kim S. H. Wee J. J. Kim Y. S. Lee H. J. (2002). Inhibitory effect of tumor cell proliferation and induction of G2/M cell cycle arrest by panaxytriol. Planta Med. 68 (2), 119–122. 10.1055/s-2002-20240 11859460

[B78] Kim J. Y. Yu S. J. Oh H. J. Lee J. Y. Kim Y. Sohn J. (2011). Panaxydol induces apoptosis through an increased intracellular calcium level, activation of JNK and p38 MAPK and NADPH oxidase-dependent generation of reactive oxygen species. Apoptosis 16 (4), 347–358. 10.1007/s10495-010-0567-8 21190085

[B79] Kim Y. S. Li X. F. Kang K. H. Ryu B. Kim S. K. (2014). Stigmasterol isolated from marine microalgae Navicula incerta induces apoptosis in human hepatoma HepG2 cells. BMB Rep. 47 (8), 433–438. 10.5483/bmbrep.2014.47.8.153 24286323 PMC4206714

[B80] Lahiani M. H. Eassa S. Parnell C. Nima Z. Ghosh A. Biris A. S. (2017). Carbon nanotubes as carriers of Panax ginseng metabolites and enhancers of ginsenosides Rb1 and Rg1 anti-cancer activity. Nanotechnology 28 (1), 015101. 10.1088/0957-4484/28/1/015101 27893436

[B81] Lamouille S. Xu J. Derynck R. (2014). Molecular mechanisms of epithelial-mesenchymal transition. Nat. Rev. Mol. Cell Biol. 15 (3), 178–196. 10.1038/nrm3758 24556840 PMC4240281

[B82] Laumont C. M. Banville A. C. Gilardi M. Hollern D. P. Nelson B. H. (2022). Tumour-infiltrating B cells: Immunological mechanisms, clinical impact and therapeutic opportunities. Nat. Rev. Cancer 22, 414–430. 10.1038/s41568-022-00466-1 35393541 PMC9678336

[B83] Le C. F. Kailaivasan T. H. Chow S. C. Abdullah Z. Ling S. K. Fang C. M. (2017). Phytosterols isolated from Clinacanthus nutans induce immunosuppressive activity in murine cells. Int. Immunopharmacol. 44, 203–210. 10.1016/j.intimp.2017.01.013 28119186

[B84] Lee D. Y. Park C. W. Lee S. J. Park H. R. Kim S. H. Son S. U. (2019). Anti-cancer effects of Panax ginseng berry polysaccharides via activation of immune-related cells. Front. Pharmacol. 10, 1411. 10.3389/fphar.2019.01411 32038228 PMC6988799

[B85] Lee H. Lee S. Jeong D. Kim S. J. (2018). Ginsenoside Rh2 epigenetically regulates cell-mediated immune pathway to inhibit proliferation of MCF-7 breast cancer cells. J. Ginseng Res. 42 (4), 455–462. 10.1016/j.jgr.2017.05.003 30337805 PMC6187096

[B86] Lee S. Y. Kim G. T. Roh S. H. Song J. S. Kim H. J. Hong S. S. (2009). Proteomic analysis of the anti-cancer effect of 20S-ginsenoside Rg3 in human colon cancer cell lines. Biosci. Biotechnol. Biochem. 73 (4), 811–816. 10.1271/bbb.80637 19352032

[B87] Lee Y. Park A. Park Y. J. Jung H. Kim T. D. Noh J. Y. (2022). Ginsenoside 20(R)-Rg3 enhances natural killer cell activity by increasing activating receptor expression through the MAPK/ERK signaling pathway. Int. Immunopharmacol. 107, 108618. 10.1016/j.intimp.2022.108618 35219164

[B88] Li B. Qu G. (2019). Inhibition of the hypoxia-induced factor-1α and vascular endothelial growth factor expression through ginsenoside Rg3 in human gastric cancer cells. J. Cancer Res. Ther. 15 (7), 1642–1646. 10.4103/jcrt.JCRT_77_17 31939450

[B89] Li G G. Xie H. Cao X. Ma C. Li Y. Chen L. (2022). Ginsenoside Rg1 exerts antiapoptotic effects on nonalcoholic fatty liver cells by downregulating the expression of SGPL1. Mol. Med. Rep. 25 (5), 178. 10.3892/mmr.2022.12694 35322862 PMC8972265

[B90] Li H. Gu L. Zhong Y. Chen Y. Zhang L. Zhang A. R. (2016a). Administration of polysaccharide from Panax notoginseng prolonged the survival of H22 tumor-bearing mice. Onco. Targets. Ther. 9, 3433–3441. 10.2147/OTT.S79427 27354815 PMC4907734

[B91] Li H. Zhang J. Zhang H. Tao Z. Yang J. Kang T. (2016b). Enzymatic digestion of total ginseng protein and determination of amino acid content. Mod. Chin. Med. 18 (01), 72–75+81.

[B92] Li J. Huang Q. Yao Y. Ji P. Mingyao E. Chen J. (2022a). Biotransformation, pharmacokinetics, and pharmacological activities of ginsenoside Rd against multiple diseases. Front. Pharmacol. 13, 909363. 10.3389/fphar.2022.909363 35928281 PMC9343777

[B93] Li J. Qi Y. (2019). Ginsenoside Rg3 inhibits cell growth, migration and invasion in Caco-2 cells by downregulation of lncRNA CCAT1. Exp. Mol. Pathol. 106, 131–138. 10.1016/j.yexmp.2019.01.003 30633886

[B94] Li J. Zhao Y. Xu N. Xing S. Shu Z. Pang B. (2022b). Pharmacological effects of ginseng protein. Special Wild Econ. Animal Plant Res. 44 (02), 121–126.

[B95] Li M. Tang D. Yang T. Qian D. Xu R. (2021a). Apoptosis triggering, an important way for natural products from herbal medicines to treat pancreatic cancers. Front. Pharmacol. 12, 796300. 10.3389/fphar.2021.796300 35222011 PMC8863938

[B96] Li M. Wang X. Wang Y. Bao S. Chang Q. Liu L. (2021b). Strategies for remodeling the tumor microenvironment using active ingredients of ginseng-A promising approach for cancer therapy. Front. Pharmacol. 12, 797634. 10.3389/fphar.2021.797634 35002732 PMC8727883

[B97] Li M. Zhang D. Cheng J. Liang J. Yu F. (2019). Retracted: Ginsenoside Rh2 inhibits proliferation but promotes apoptosis and autophagy by down-regulating microRNA-638 in human retinoblastoma cells. Exp. Mol. Pathol. 108, 17–23. 10.1016/j.yexmp.2019.03.004 30853612

[B98] Li Q. Jiang C. Zhang L. Qiu W. Meng X. (2012). Apoptosis of human hepatocellular carcinoma cells SMMC-7721 induced by β-sitosterol and dousterol. Lishizhen Med. Materia Medica Res. 23 (05), 1173–1175.

[B99] Li Q. Li B. Dong C. Wang Y. Li Q. (2017). 20(S)-Ginsenoside Rh2 suppresses proliferation and migration of hepatocellular carcinoma cells by targeting EZH2 to regulate CDKN2A-2B gene cluster transcription. Eur. J. Pharmacol. 815, 173–180. 10.1016/j.ejphar.2017.09.023 28928088

[B100] Li W. F. Chen L. R. Gong X. J. Li Z. N. Li K. K. (2013). Synthesis of esters of ginsenoside metabolite M1 and their cytotoxicity on MGC80-3 cells. Molecules 18 (4), 3689–3702. 10.3390/molecules18043689 23529029 PMC6270463

[B101] Li X. Lin Z. Zhang B. Guo L. Liu S. Li H. (2016). β-elemene sensitizes hepatocellular carcinoma cells to oxaliplatin by preventing oxaliplatin-induced degradation of copper transporter 1. Sci. Rep. 6, 21010. 10.1038/srep21010 26867799 PMC4751482

[B102] Li X. Tsauo J. Geng C. Zhao H. Lei X. Li X. (2018). Ginsenoside Rg3 decreases NHE1 expression via inhibiting EGF-EGFR-ERK1/2-HIF-1 alpha pathway in hepatocellular carcinoma: A novel antitumor mechanism. Am. J. Chin. Med. 46 (8), 1915–1931. 10.1142/S0192415X18500969 30525897

[B103] Li Y Y. He F. Zhang Y. Pan Z. (2022). Apatinib and ginsenoside-Rb1 synergetically control the growth of hypopharyngeal carcinoma cells. Dis. Markers 2022, 3833489. 10.1155/2022/3833489 35069931 PMC8776476

[B104] Liang Q. Yang J. He J. Chen X. Zhang H. Jia M. (2020). Stigmasterol alleviates cerebral ischemia/reperfusion injury by attenuating inflammation and improving antioxidant defenses in rats. Biosci. Rep. 40 (4), BSR20192133. 10.1042/BSR20192133 32149332 PMC7160377

[B105] Lin H. Piao B. Li S. (2002). Summary of phase Ⅱ clinical trial of shenyi capsule in the treatment of lung cancer. Chin. J. Clin. Oncol. 1 (04), 52–55.

[B106] Ling S. Li J. Shan Q. Dai H. Lu D. Wen X. (2017). USP22 mediates the multidrug resistance of hepatocellular carcinoma via the SIRT1/AKT/MRP1 signaling pathway. Mol. Oncol. 11 (6), 682–695. 10.1002/1878-0261.12067 28417539 PMC5467492

[B107] Liu G. W. Liu Y. H. Jiang G. S. Ren W. D. (2018). The reversal effect of Ginsenoside Rh2 on drug resistance in human colorectal carcinoma cells and its mechanism. Hum. Cell. 31 (3), 189–198. 10.1007/s13577-017-0189-3 29582366

[B108] Liu G. Zhang J. Sun F. Ma J. Qi X. (2022). Ginsenoside Rg2 attenuated trastuzumab-induced cardiotoxicity in rats. Biomed. Res. Int. 2022, 8866660. 10.1155/2022/8866660 35071601 PMC8769853

[B109] Liu H. Zhao J. Fu R. Zhu C. Fan D. (2019). The ginsenoside Rk3 exerts anti-esophageal cancer activity *in vitro* and *in vivo* by mediating apoptosis and autophagy through regulation of the PI3K/Akt/mTOR pathway. PLoS One 14 (5), e0216759. 10.1371/journal.pone.0216759 31091245 PMC6519821

[B110] Liu J. Wang Y. Yu Z. Lv G. Huang X. Lin H. (2022). Functional mechanism of ginsenoside compound K on tumor growth and metastasis. Integr. Cancer Ther. 21, 1. 10.1177/15347354221101203 PMC9152193 35615883

[B111] Liu S. Chen M. Li P. Wu Y. Chang C. Qiu Y. (2015). Ginsenoside rh2 inhibits cancer stem-like cells in skin squamous cell carcinoma. Cell. Physiol. biochem. 36 (2), 499–508. 10.1159/000430115 25966742

[B112] Liu T. Duo L. Duan P. (2018). Ginsenoside Rg3 sensitizes colorectal cancer to radiotherapy through downregulation of proliferative and angiogenic biomarkers. Evid. Based. Complement. Altern. Med. 2018, 1580427. 10.1155/2018/1580427 PMC5878898 29743919

[B113] Liu Y. Fan D. (2019). Ginsenoside Rg5 induces G2/M phase arrest, apoptosis and autophagy via regulating ROS-mediated MAPK pathways against human gastric cancer. Biochem. Pharmacol. 168, 285–304. 10.1016/j.bcp.2019.07.008 31301277

[B114] Liu Y. P. Zheng C. C. Huang Y. N. He M. L. Xu W. W. Li B. (2021). Molecular mechanisms of chemo- and radiotherapy resistance and the potential implications for cancer treatment. MedComm 2 (3), 315–340. 10.1002/mco2.55 34766149 PMC8554658

[B115] Liu Z. Liu T. Li W. Li J. Wang C. Zhang K. (2021). Insights into the antitumor mechanism of ginsenosides Rg3. Mol. Biol. Rep. 48 (3), 2639–2652. 10.1007/s11033-021-06187-2 33661439

[B116] Lu H. Yin H. Qu L. Ma X. Fu R. Fan D. (2022). Ginsenoside Rk1 regulates glutamine metabolism in hepatocellular carcinoma through inhibition of the ERK/c-Myc pathway. Food Funct. 13 (7), 3793–3811. 10.1039/d1fo03728e 35316310

[B117] Lu M. Fei Z. Zhang G. (2018). Synergistic anticancer activity of 20(S)-Ginsenoside Rg3 and Sorafenib in hepatocellular carcinoma by modulating PTEN/Akt signaling pathway. Biomed. Pharmacother. 97, 1282–1288. 10.1016/j.biopha.2017.11.006 29156516

[B118] Lu S. Zhang Y. Li H. Zhang J. Ci Y. Han M. (2020). Ginsenoside Rb1 can ameliorate the key inflammatory cytokines TNF-alpha and IL-6 in a cancer cachexia mouse model. BMC Complement. Med. Ther. 20 (1), 11. 10.1186/s12906-019-2797-9 32020864 PMC7076885

[B119] Luo L. Shi Y. Jiang Y. Zhan J. Tan L. Chen N. (2017). Advances in the study of active ingredients and their mechanisms of anti-tumor effects of ginseng. Chin. Traditional Herb. Drugs 48 (03), 582–596.

[B120] Luo X. Wang H. Ji D. (2021). Carbon nanotubes (CNT)-loaded ginsenosides Rb3 suppresses the PD-1/PD-L1 pathway in triple-negative breast cancer. Aging (Albany NY) 13 (13), 17177–17189. 10.18632/aging.203131 34111025 PMC8312428

[B121] Ma H. Yu Y. Wang M. Li Z. Xu H. Tian C. (2019). Correlation between microbes and colorectal cancer: Tumor apoptosis is induced by sitosterols through promoting gut microbiota to produce short-chain fatty acids. Apoptosis 24 (1-2), 168–183. 10.1007/s10495-018-1500-9 30506375

[B122] Ma J. Gao G. Lu H. Fang D. Li L. Wei G. (2019). Reversal effect of ginsenoside Rh2 on oxaliplatin-resistant colon cancer cells and its mechanism. Exp. Ther. Med. 18 (1), 630–636. 10.3892/etm.2019.7604 31258699 PMC6566025

[B123] Ma L. Miao D. Lee J. J. Li T. Chen Y. Su G. (2021). Synthesis and biological evaluation of heterocyclic ring-fused dammarane-type ginsenoside derivatives as potential anti-tumor agents. Bioorg. Chem. 116, 105365. 10.1016/j.bioorg.2021.105365 34563998

[B124] Mahmoud A. A. El-Sayed W. M. (2019). The anti-proliferative activity of anisosciadone: A new guaiane sesquiterpene from anisosciadium lanatum. Anticancer. Agents Med. Chem. 19 (9), 1114–1119. 10.2174/1871520619666190308112732 30848216

[B125] Mao Q. Zhang P. H. Yang J. Xu J. D. Kong M. Shen H. (2016). iTRAQ-based proteomic analysis of ginsenoside F2 on human gastric carcinoma cells SGC7901. Evid. Based. Complement. Altern. Med. 2016, 2635483. 10.1155/2016/2635483 PMC5088344 27829861

[B126] Mizushima N. Levine B. Cuervo A. M. Klionsky D. J. (2008). Autophagy fights disease through cellular self-digestion. Nature 451 (7182), 1069–1075. 10.1038/nature06639 18305538 PMC2670399

[B127] Mylod E. Lysaght J. Conroy M. J. (2022). Natural killer cell therapy: A new frontier for obesity-associated cancer. Cancer Lett. 535, 215620. 10.1016/j.canlet.2022.215620 35283210

[B128] Nakhjavani M. Smith E. Townsend A. R. Price T. J. Hardingham J. E. (2020). Anti-angiogenic properties of ginsenoside Rg3. Molecules 25 (21), E4905. 10.3390/molecules25214905 33113992 PMC7660320

[B129] Nisha R. Kumar P. Gautam A. K. Bera H. Bhattacharya B. Parashar P. (2021). Assessments of *in vitro* and *in vivo* antineoplastic potentials of beta-sitosterol-loaded PEGylated niosomes against hepatocellular carcinoma. J. Liposome Res. 31 (3), 304–315. 10.1080/08982104.2020.1820520 32901571

[B130] Pak J. N. Jung J. H. Park J. E. Hwang J. Lee H. J. Shim B. S. (2020). p53 dependentLGR5 inhibition and caspase 3 activation are critically involved in apoptotic effect of compound K and its combination therapy potential inHCT116 cells. Phytother. Res. 34 (10), 2745–2755. 10.1002/ptr.6717 32403193

[B131] Pandey P. Bajpai P. Siddiqui M. H. Sayyed U. Tiwari R. Shekh R. (2019). Elucidation of the chemopreventive role of stigmasterol against Jab1 in gall bladder carcinoma. Endocr. Metab. Immune Disord. Drug Targets 19 (6), 826–837. 10.2174/1871530319666190206124120 30727937

[B132] Pandith H. Zhang X. Thongpraditchote S. Wongkrajang Y. Gritsanapan W. Baek S. J. (2013). Effect of Siam weed extract and its bioactive component scutellarein tetramethyl ether on anti-inflammatory activity through NF-κB pathway. J. Ethnopharmacol. 147 (2), 434–441. 10.1016/j.jep.2013.03.033 23535395

[B133] Paramasivam A. Priyadharsini J. V. (2021). RNA N6-methyladenosine: A new player in autophagy-mediated anti-cancer drug resistance. Br. J. Cancer 124 (10), 1621–1622. 10.1038/s41416-021-01314-z 33723389 PMC8110764

[B134] Phi L. Wijaya Y. T. Sari I. N. Kim K. S. Yang Y. G. Lee M. W. (2019). 20(R)-Ginsenoside Rg3 influences cancer stem cell properties and the epithelial-mesenchymal transition in colorectal cancer via the SNAIL signaling Axis. Onco. Targets. Ther. 12, 10885–10895. 10.2147/OTT.S219063 31849492 PMC6912006

[B135] Phi L. Wijaya Y. T. Sari I. N. Yang Y. G. Lee Y. K. Kwon H. Y. (2018). The anti-metastatic effect of ginsenoside Rb2 in colorectal cancer in an EGFR/SOX2-dependent manner. Cancer Med. 7 (11), 5621–5631. 10.1002/cam4.1800 30264477 PMC6246932

[B136] Pu Z. Ge F. Wang Y. Jiang Z. Zhu S. Qin S. (2021). Ginsenoside-Rg3 inhibits the proliferation and invasion of hepatoma carcinoma cells via regulating long non-coding RNA HOX antisense intergenic. Bioengineered 12 (1), 2398–2409. 10.1080/21655979.2021.1932211 34130594 PMC8806740

[B137] Qi Z. Chen L. Li Z. Shao Z. Qi Y. Gao K. (2019). Immunomodulatory effects of (24r)-pseudo-ginsenoside HQ and (24S)-Pseudo-Ginsenoside HQ on cyclophosphamide-induced immunosuppression and their anti-tumor effects study. Int. J. Mol. Sci. 20 (4), E836. 10.3390/ijms20040836 30769948 PMC6413033

[B138] Qian J. Li J. Jia J. G. Jin X. Yu D. J. Guo C. X. (2016). Ginsenoside-Rh2 inhibits proliferation and induces apoptosis of human gastric cancer SGC-7901 side population cells. Asian pac. J. Cancer Prev. 17 (4), 1817–1821. 10.7314/apjcp.2016.17.4.1817 27221858

[B139] Qiu R. Qian F. Wang X. Li H. Wang L. (2019). Targeted delivery of 20(S)-ginsenoside Rg3-based polypeptide nanoparticles to treat colon cancer. Biomed. Microdevices 21 (1), 18. 10.1007/s10544-019-0374-0 30783757

[B140] Qu L. Ma X. Fan D. (2021). Ginsenoside Rk3 suppresses hepatocellular carcinoma development through targeting the gut-liver Axis. J. Agric. Food Chem. 69 (35), 10121–10137. 10.1021/acs.jafc.1c03279 34415764

[B141] Qu Y. Li S. Zhang S. Gao J. Lin Z. Yin Y. (2021). A network pharmacological study on the prevention of postoperative recurrence and metastasis of colorectal cancer with Radix Codonopsis pilosulae and Radix et Rhizoma alba. Anti-Tumor Pharm. 11 (06), 707–719.

[B142] Raj R. K. D E. S R. (2020). β-Sitosterol-assisted silver nanoparticles activates Nrf2 and triggers mitochondrial apoptosis via oxidative stress in human hepatocellular cancer cell line. J. Biomed. Mat. Res. A 108 (9), 1899–1908. 10.1002/jbm.a.36953 32319188

[B143] Raj S. Jayaraj R. Kodiveri M. G. (2022). Chemical profiling and evaluation of antioxidant and anticancer potential of tuber crop Amorphophallus commutatus var. wayanadensis. Plant Foods Hum. Nutr. 77 (1), 68–76. 10.1007/s11130-021-00942-3 34977995

[B144] Rajavel T. Packiyaraj P. Suryanarayanan V. Singh S. K. Ruckmani K. Pandima D. K. (2018). β-Sitosterol targets Trx/Trx1 reductase to induce apoptosis in A549 cells via ROS mediated mitochondrial dysregulation and p53 activation. Sci. Rep. 8 (1), 2071. 10.1038/s41598-018-20311-6 29391428 PMC5794769

[B145] Rather R. A. Bhagat M. (2020). Quercetin as an innovative therapeutic tool for cancer chemoprevention: Molecular mechanisms and implications in human health. Cancer Med. 9 (24), 9181–9192. 10.1002/cam4.1411 31568659 PMC7774748

[B146] Ren Y. (2019). Extraction of ginseng protein and its antitumor effect on breast cancer MCF-7 cells. Changchun: Changchun University of Chinese Medicine, 59.

[B147] Ren Z. Chen X. Hong L. Zhao X. Cui G. Li A. (2020). Nanoparticle conjugation of ginsenoside Rg3 inhibits hepatocellular carcinoma development and metastasis. Small 16 (2), e1905233. 10.1002/smll.201905233 31814271

[B148] Riabov V. Gudima A. Wang N. Mickley A. Orekhov A. Kzhyshkowska J. (2014). Role of tumor associated macrophages in tumor angiogenesis and lymphangiogenesis. Front. Physiol. 5, 75. 10.3389/fphys.2014.00075 24634660 PMC3942647

[B149] Roy R. K. Yadav R. Sharma U. Wasson M. K. Sharma A. Tanwar P. (2022). Impact of noncoding RNAs on cancer directed immune therapies: Now then and forever. Int. J. Cancer 151, 981–992. 10.1002/ijc.34060 35489027

[B150] Ryan K. M. (2011). p53 and autophagy in cancer: guardian of the genome meets guardian of the proteome. Eur. J. Cancer. 47 (1), 44–50. 10.1016/j.ejca.2010.10.020 21112207

[B151] Sabeva N. S. Mcphaul C. M. Li X. Cory T. J. Feola D. J. Graf G. A. (2011). Phytosterols differentially influence ABC transporter expression, cholesterol efflux and inflammatory cytokine secretion in macrophage foam cells. J. Nutr. Biochem. 22 (8), 777–783. 10.1016/j.jnutbio.2010.07.002 21111593 PMC3075387

[B152] Salehi B. Quispe C. Sharifi-Rad J. Cruz-Martins N. Nigam M. Mishra A. P. (2020). Phytosterols: From preclinical evidence to potential clinical applications. Front. Pharmacol. 11, 599959. 10.3389/fphar.2020.599959 33519459 PMC7841260

[B153] Shang H. Li L. Ma L. Tian Y. Jia H. Zhang T. (2020). Design and synthesis of molecular hybrids of Sophora alkaloids and cinnamic acids as potential antitumor agents. Molecules 25 (5), E1168. 10.3390/molecules25051168 32150948 PMC7179170

[B154] Sharmila R. Sindhu G. (2017). Modulation of angiogenesis, proliferative response and apoptosis by beta-sitosterol in rat model of renal carcinogenesis. Indian J. Clin. biochem. 32 (2), 142–152. 10.1007/s12291-016-0583-8 28428688 PMC5382068

[B155] Shathviha P. C. Ezhilarasan D. Rajeshkumar S. Selvaraj J. (2021). β-Sitosterol mediated silver nanoparticles induce cytotoxicity in human colon cancer HT-29 cells. Avicenna J. Med. Biotechnol. 13 (1), 42–46. 10.18502/ajmb.v13i1.4577 33680372 PMC7903430

[B156] Shen M. Hu Y. Yang Y. Wang L. Yang X. Wang B. (2019). Betulinic acid induces ROS-dependent apoptosis and S-phase Arrest by inhibiting the NF-κB pathway in human multiple myeloma. Oxid. Med. Cell. Longev. 2019, 5083158. 10.1155/2019/5083158 31281581 PMC6590575

[B157] Shi Q. Shi X. Zuo G. Xiong W. Li H. Guo P. (2016). Anticancer effect of 20(S)-ginsenoside Rh2 on HepG2 liver carcinoma cells: Activating GSK-3β and degrading β-catenin. Oncol. Rep. 36 (4), 2059–2070. 10.3892/or.2016.5033 27573179

[B158] Shi Z. Y. Zeng J. Z. Wong A. (2019). Chemical structures and pharmacological profiles of ginseng saponins. Molecules 24 (13), E2443. 10.3390/molecules24132443 31277214 PMC6651355

[B159] Shin E. J. Choi H. K. Sung M. J. Park J. H. Chung M. Y. Chung S. (2018). Anti-tumour effects of beta-sitosterol are mediated by AMPK/PTEN/HSP90 axis in AGS human gastric adenocarcinoma cells and xenograft mouse models. Biochem. Pharmacol. 152, 60–70. 10.1016/j.bcp.2018.03.010 29559312

[B160] Shin N. Lee H. J. Sim D. Y. Im E. Park J. E. Park W. Y. (2021). Apoptotic effect of compound K in hepatocellular carcinoma cells via inhibition of glycolysis and Akt/mTOR/c-Myc signaling. Phytother. Res. 35 (7), 3812–3820. 10.1002/ptr.7087 33856720

[B161] Srijiwangsa P. Ponnikorn S. Na-Bangchang K. (2018). Effect of beta-Eudesmol on NQO1 suppression-enhanced sensitivity of cholangiocarcinoma cells to chemotherapeutic agents. BMC Pharmacol. Toxicol. 19 (1), 32. 10.1186/s40360-018-0223-4 29914576 PMC6006851

[B162] Su J. Zhang F. Li X. Liu Z. (2019). Osthole promotes the suppressive effects of cisplatin on NRF2 expression to prevent drug-resistant cervical cancer progression. Biochem. Biophys. Res. Commun. 514 (2), 510–517. 10.1016/j.bbrc.2019.04.021 31056260

[B163] Su P. Ahmad B. Zou K. Zou L. (2020). β-Elemene enhances the chemotherapeutic effect of 5-fluorouracil in triple-negative breast cancer via PI3K/AKT, RAF-MEK-ErK, and NF-κB signaling pathways. Onco. Targets. Ther. 13, 5207–5222. 10.2147/OTT.S242820 32606741 PMC7294576

[B164] Subramanian S. Steer C. J. (2019). Special issue: MicroRNA regulation in health and disease. Genes (Basel) 10 (6), E457. 10.3390/genes10060457 31208024 PMC6628077

[B165] Sun D. Zou Y. Song L. Han S. Yang H. Chu D. (2022). A cyclodextrin-based nanoformulation achieves co-delivery of ginsenoside Rg3 and quercetin for chemo-immunotherapy in colorectal cancer. Acta Pharm. Sin. B 12 (1), 378–393. 10.1016/j.apsb.2021.06.005 35127393 PMC8799998

[B166] Sun M. Y. Song Y. N. Zhang M. Zhang C. Y. Zhang L. J. Zhang H. (2019). Ginsenoside Rg3 inhibits the migration and invasion of liver cancer cells by increasing the protein expression of ARHGAP9. Oncol. Lett. 17 (1), 965–973. 10.3892/ol.2018.9701 30655855 PMC6313058

[B167] Tang J. (2021). Screening of effective components of the Chinese medicine Sanleng and its effect on the proliferation and apoptosis of gastric cancer MGC-803 cells. Hengyang: Nanhua University, 80.

[B168] Tang X. Liu J. Li L. Du R. (2012). Pharmacological effects of organic acids in traditional Chinese medicine and their application in cardiovascular diseases. Chin. J. Exp. Traditional Med. Formulae 18 (05), 243–246.

[B169] Tang Y. C. Zhang Y. Zhou J. Zhi Q. Wu M. Y. Gong F. R. (2018). Ginsenoside Rg3 targets cancer stem cells and tumor angiogenesis to inhibit colorectal cancer progression *in vivo* . Int. J. Oncol. 52 (1), 127–138. 10.3892/ijo.2017.4183 29115601 PMC5743384

[B170] Tian Y. Z. Liu Y. P. Tian S. C. Ge S. Y. Wu Y. J. Zhang B. L. (2020). Antitumor activity of ginsenoside Rd in gastric cancer via up-regulation of Caspase-3 and Caspase-9. Pharmazie 75 (4), 147–150. 10.1691/ph.2020.9931 32295691

[B171] Tovey F. I. (2015). Role of dietary phospholipids and phytosterols in protection against peptic ulceration as shown by experiments on rats. World J. Gastroenterol. 21 (5), 1377–1384. 10.3748/wjg.v21.i5.1377 25663757 PMC4316080

[B172] Wang B. Xu Q. Zhou C. Lin Y. (2021). Liposomes co-loaded with ursolic acid and ginsenoside Rg3 in the treatment of hepatocellular carcinoma. Acta Biochim. Pol. 68 (4), 711–715. 10.18388/abp.2020_5608 34730903

[B173] Wang C. Z. Hou L. Wan J. Y. Yao H. Yuan J. Zeng J. (2020). Ginseng berry polysaccharides on inflammation-associated colon cancer: Inhibiting T-cell differentiation, promoting apoptosis, and enhancing the effects of 5-fluorouracil. J. Ginseng Res. 44 (2), 282–290. 10.1016/j.jgr.2018.12.010 32148410 PMC7031751

[B174] Wang G. Y. Zhang L. Geng Y. D. Wang B. Feng X. J. Chen Z. L. (2022). β-Elemene induces apoptosis and autophagy in colorectal cancer cells through regulating the ROS/AMPK/mTOR pathway. Chin. J. Nat. Med. 20 (1), 9–21. 10.1016/S1875-5364(21)60118-8 35101253

[B175] Wang J. Xu C. Chen Y. Shao L. Li T. Fan X. (2021). β-elemene enhances the antitumor activity of erlotinib by inducing apoptosis through AMPK and MAPK pathways in TKI-resistant H1975 lung cancer cells. J. Cancer. 12 (8), 2285–2294. 10.7150/jca.53382 33758606 PMC7974887

[B176] Wang W. Zhong W. Yuan J. Yan C. Hu S. Tong Y. (2015). Involvement of Wnt/β-catenin signaling in the mesenchymal stem cells promote metastatic growth and chemoresistance of cholangiocarcinoma. Oncotarget 6 (39), 42276–42289. 10.18632/oncotarget.5514 26474277 PMC4747224

[B177] Wang X. He R. Geng L. Yuan J. Fan H. (2022). Ginsenoside Rg3 alleviates cisplatin resistance of gastric cancer cells through inhibiting SOX2 and the PI3K/Akt/mTOR signaling Axis by up-regulating miR-429. Front. Genet. 13, 823182. 10.3389/fgene.2022.823182 35309116 PMC8927288

[B178] Wang X. Sun Y. Y. Qu F. Z. Su G. Y. Zhao Y. Q. (2019). 4-XL-PPD, a novel ginsenoside derivative, as potential therapeutic agents for gastric cancer shows anti-cancer activity via inducing cell apoptosis medicated generation of reactive oxygen species and inhibiting migratory and invasive. Biomed. Pharmacother. 118, 108589. 10.1016/j.biopha.2019.01.050 31382131

[B179] Wang Y. S. Chen C. Zhang S. Y. Li Y. Jin Y. H. (2021). (20S ginsenoside Rh2 inhibits STAT3/VEGF signaling by targeting annexin A2. Int. J. Mol. Sci. 22 (17), 9289. 10.3390/ijms22179289 34502195 PMC8431727

[B180] Wang Y. S. Zhu H. Li H. Li Y. Zhao B. Jin Y. H. (2019). Ginsenoside compound K inhibits nuclear factor-kappa B by targeting Annexin A2. J. Ginseng Res. 43 (3), 452–459. 10.1016/j.jgr.2018.04.002 31308817 PMC6606818

[B181] Wang Z. Zhan Y. Xu J. Wang Y. Sun M. Chen J. (2020). β-Sitosterol reverses multidrug resistance via BCRP suppression by inhibiting the p53-MDM2 interaction in colorectal cancer. J. Agric. Food Chem. 68 (12), 3850–3858. 10.1021/acs.jafc.0c00107 32167760

[B182] Wilkins L. R. Brautigan D. L. Wu H. Yarmohammadi H. Kubicka E. Serbulea V. (2017). Cinnamic acid derivatives enhance the efficacy of transarterial embolization in a rat model of hepatocellular carcinoma. Cardiovasc. Interv. Radiol. 40 (3), 430–437. 10.1007/s00270-016-1515-y PMC5520990 27872984

[B183] Wu C. Zeng M. H. Liao G. Qian K. Li H. (2020). Neuropilin-1 interacts with fibronectin-1 to promote epithelial-mesenchymal transition progress in gastric cancer. Onco. Targets. Ther. 13, 10677–10687. 10.2147/OTT.S275327 33116644 PMC7585825

[B184] Wu H. C. Hu Q. R. Luo T. Wei W. C. Wu H. J. Li J. (2021). The immunomodulatory effects of ginsenoside derivative Rh2-O on splenic lymphocytes in H22 tumor-bearing mice is partially mediated by TLR4. Int. Immunopharmacol. 101 (1), 108316. 10.1016/j.intimp.2021.108316 34768129

[B185] Wu J. (2017). Effect of ginsenoside triol on PXR-CYP3A4 regulatory pathway in HepG2 cells and its mechanism. Nanchang: Nanchang University, 92.

[B186] Wu J. Tang X. Shi Y. Ma C. Zhang H. Zhang J. (2022). Crosstalk of LncRNA HOTAIR and SP1-mediated repression of PDK1 contributes to beta-Elemene-inhibited proliferation of hepatocellular carcinoma cells. J. Ethnopharmacol. 283, 114456. 10.1016/j.jep.2021.114456 34333105

[B187] Wu J. Yao N. Hu Q. Liu M. Zhang H. Xiong Y. (2019). Effect of panaxytriol on cytochrome P450 3A4 via the pregnane X receptor regulatory pathway. Phytother. Res. 33 (4), 968–975. 10.1002/ptr.6290 30653754

[B188] Wu K. Zou L. Lei X. Yang X. (2022). Roles of ABCA1 in cancer. Oncol. Lett. 24 (4), 349. 10.3892/ol.2022.13469 36072007 PMC9434721

[B189] Wu R. Ru Q. Chen L. Ma B. Li C. (2014). Stereospecificity of ginsenoside Rg3 in the promotion of cellular immunity in hepatoma H22-bearing mice. J. Food Sci. 79 (7), H1430–H1435. 10.1111/1750-3841.12518 25041540

[B190] Xia T. Zhang B. Li Y. Fang B. Zhu X. Xu B. (2020). New insight into 20(S)-ginsenoside Rh2 against T-cell acute lymphoblastic leukemia associated with the gut microbiota and the immune system. Eur. J. Med. Chem. 203, 112582. 10.1016/j.ejmech.2020.112582 32682197

[B191] Xiao S. Lin Z. Wang X. Lu J. Zhao Y. (2020). Synthesis and cytotoxicity evaluation of panaxadiol derivatives. Chem. Biodivers. 17 (1), e1900516. 10.1002/cbdv.201900516 31725193

[B192] Xiaomeng F. Lei L. Jinghong A. Juan J. Qi Y. Dandan Y. (2020). Treatment with β-elemene combined with paclitaxel inhibits growth, migration, and invasion and induces apoptosis of ovarian cancer cells by activation of STAT-NF-κB pathway. Braz. J. Med. Biol. Res. 53 (6), e8885. 10.1590/1414-431x20208885 32401925 PMC7228545

[B193] Xie J. Luo S. Mi H. Du Y. Bao G. Zhou J. (2019). Intake consumption of ginsenoside Rg3, profiling of selected cytokines, and development of rectal polyps. Cancer Manag. Res. 11, 4059–4064. 10.2147/CMAR.S197097 31190981 PMC6511619

[B194] Xiong H. Ni Z. He J. Jiang S. Li X. He J. (2017). LncRNA HULC triggers autophagy via stabilizing Sirt1 and attenuates the chemosensitivity of HCC cells. Oncogene 36 (25), 3528–3540. 10.1038/onc.2016.521 28166203

[B195] Xu J. F. Wan Y. Tang F. Chen L. Yang Y. Xia J. (2021). Emerging significance of ginsenosides as potentially reversal agents of chemoresistance in cancer therapy. Front. Pharmacol. 12, 720474. 10.3389/fphar.2021.720474 34975466 PMC8719627

[B196] Yang J. Yuan D. Xing T. Su H. Zhang S. Wen J. (2016). Ginsenoside Rh2 inhibiting HCT116 colon cancer cell proliferation through blocking PDZ-binding kinase/T-LAK cell-originated protein kinase. J. Ginseng Res. 40 (4), 400–408. 10.1016/j.jgr.2016.03.007 27746693 PMC5052442

[B197] Yang L L. Li J. Hu Z. Fan X. Cai T. Hengli Z. (2020). A systematic review of the mechanisms underlying treatment of gastric precancerous lesions by traditional Chinese medicine. Evid. Based. Complement. Altern. Med. 2020, 9154738. 10.1155/2020/9154738 PMC7212333 32454874

[B198] Yang L L. Zhang X. Y. Li K. Li A. P. Yang W. D. Yang R. (2019). Protopanaxadiol inhibits epithelial-mesenchymal transition of hepatocellular carcinoma by targeting STAT3 pathway. Cell Death Dis. 10 (9), 630. 10.1038/s41419-019-1733-8 31431619 PMC6702205

[B199] Yang M. Wang C. C. Wang W. L. Xu J. P. Wang J. Zhang C. H. (2020). Saposhnikovia divaricata-an ethnopharmacological, phytochemical and pharmacological review. Chin. J. Integr. Med. 26 (11), 873–880. 10.1007/s11655-020-3091-x 32328867 PMC7176574

[B200] Yang P. Song L. Zhang L. Zhu H. Wang D. (2019). Effects of panaxynol combined with gemcitabine on the differentiation and activity of pancreatic cancer stem cells. J. Jiangsu Univ. Ed. 29 (03), 221–225.

[B201] Yang Q. Cai N. Che D. Chen X. Wang D. (2020). Ginsenoside Rg3 inhibits the biological activity of SGC-7901. Food Sci. Nutr. 8 (8), 4151–4158. 10.1002/fsn3.1707 32884696 PMC7455926

[B202] Yang X. Zou J. Cai H. Huang X. Yang X. Guo D. (2017). Ginsenoside Rg3 inhibits colorectal tumor growth via down-regulation of C/EBPβ/NF-κB signaling. Biomed. Pharmacother. 96, 1240–1245. 10.1016/j.biopha.2017.11.092 29169725

[B203] Yao H. Wan J. Y. Zeng J. Huang W. H. Sava-Segal C. Li L. (2018). Effects of compound K, an enteric microbiome metabolite of ginseng, in the treatment of inflammation associated colon cancer. Oncol. Lett. 15 (6), 8339–8348. 10.3892/ol.2018.8414 29805567 PMC5950138

[B204] Yuan Y. Wang J. Xu M. Zhang Y. Wang Z. Liang L. (2020). 20(S)-ginsenoside Rh2 as agent for the treatment of LMN-CRC via regulating epithelial-mesenchymal transition. Biosci. Rep. 40 (3), BSR20191507. 10.1042/BSR20191507 32141497 PMC7098129

[B205] Yun U. J. Lee I. H. Lee J. S. Shim J. Kim Y. N. (2020). Ginsenoside Rp1, A ginsenoside derivative, augments anti-cancer effects of actinomycin D via downregulation of an AKT-SIRT1 pathway. Cancers (Basel) 12 (3), E605. 10.3390/cancers12030605 32151067 PMC7139315

[B206] Zeng J. Ma X. Zhao Z. Chen Y. Wang J. Hao Y. (2021). Ginsenoside Rb1 lessens gastric precancerous lesions by interfering with beta-catenin/TCF4 interaction. Front. Pharmacol. 12, 682713. 10.3389/fphar.2021.682713 34594214 PMC8476751

[B207] Zhang E. Shi H. Yang L. Wu X. Wang Z. (2017). Ginsenoside Rd regulates the Akt/mTOR/p70S6K signaling cascade and suppresses angiogenesis and breast tumor growth. Oncol. Rep. 38 (1), 359–367. 10.3892/or.2017.5652 28534996

[B208] Zhang F. Li M. Wu X. Hu Y. Cao Y. Wang X. (2015). 20(S)-ginsenoside Rg3 promotes senescence and apoptosis in gallbladder cancer cells via the p53 pathway. Drug Des. devel. Ther. 9, 3969–3987. 10.2147/DDDT.S84527 PMC4539091 26309394

[B209] Zhang F. Xu H. Xia R. Yu P. Li Y. Yu X. (2021). Pseudo-ginsenoside Rh2 induces protective autophagy in hepatocellular carcinoma HepG2 cells. Recent Pat. anticancer. Drug Discov. 16 (4), 521–532. 10.2174/1574892816666210607100239 34109916

[B210] Zhang H. Yi J. K. Huang H. Park S. Kwon W. Kim E. (2022). 20 (S)-ginsenoside Rh2 inhibits colorectal cancer cell growth by suppressing the Axl signaling pathway *in vitro* and *in vivo* . J. Ginseng Res. 46 (3), 396–407. 10.1016/j.jgr.2021.07.004 35600769 PMC9120647

[B211] Zhang J. Li W. Yuan Q. Zhou J. Zhang J. Cao Y. (2019). Transcriptome analyses of the anti-proliferative effects of 20(S)-Ginsenoside Rh2 on HepG2 cells. Front. Pharmacol. 10, 1331. 10.3389/fphar.2019.01331 31780945 PMC6855211

[B212] Zhang J. Ma X. Fan D. (2022). Ginsenoside CK ameliorates hepatic lipid accumulation via activating the LKB1/AMPK pathway *in vitro* and *in vivo* . Food Funct. 13 (3), 1153–1167. 10.1039/d1fo03026d 35018944

[B213] Zhang J. Ma X. Fan D. (2021). Ginsenoside CK inhibits hypoxia-induced epithelial-mesenchymal transformation through the HIF-1α/NF-κB feedback pathway in hepatocellular carcinoma. Foods 10 (6), 1195. 10.3390/foods10061195 34073155 PMC8227303

[B214] Zhang K K. Liu Y. Wang C. Li J. Xiong L. Wang Z. (2019). Evaluation of the gastroprotective effects of 20 (S)-ginsenoside Rg3 on gastric ulcer models in mice. J. Ginseng Res. 43 (4), 550–561. 10.1016/j.jgr.2018.04.001 31695563 PMC6823781

[B215] Zhang L. (2021). Mechanism of upregulation of CYP3A4 expression by ginsenosperm triol based on the interaction of PXR, CAR with HSP90 and RXR. Nanchang: Nanchang University, 80.

[B216] Zhang R. Pan T. Xiang Y. Zhang M. Feng J. Liu S. (2020). β-Elemene reverses the resistance of p53-deficient colorectal cancer cells to 5-fluorouracil by inducing pro-death autophagy and cyclin D3-dependent cycle arrest. Front. Bioeng. Biotechnol. 8, 378. 10.3389/fbioe.2020.00378 32457882 PMC7225311

[B217] Zhang S. Wang H. Shi Z. Zhang L. Song Y. Yue W. (2007). Application of gene chip technology to study the target gene regulation of Hedyotis diffusa stigmasterol in inhibiting the growth of human hepatoma cells *in vitro* . Prog. Mod. Biomed. 1 (08), 1181–1183.

[B218] Zhang S. Zhang M. Chen J. Zhao J. Su J. Zhang X. (2020). Ginsenoside compound K regulates HIF-1α-Mediated glycolysis through Bclaf1 to inhibit the proliferation of human liver cancer cells. Front. Pharmacol. 11, 583334. 10.3389/fphar.2020.583334 33363466 PMC7753211

[B219] Zhang X. F. Li K. K. Gao L. Li S. Z. Chen K. Zhang J. B. (2015). miR-191 promotes tumorigenesis of human colorectal cancer through targeting C/EBPβ. Oncotarget 6 (6), 4144–4158. 10.18632/oncotarget.2864 25784653 PMC4414178

[B220] Zhang X. Sun K. Zhu Q. Song T. Liu Y. (2017b). Ginseng polysaccharide serves as a potential radiosensitizer through inducing apoptosis and autophagy in the treatment of osteosarcoma. Kaohsiung J. Med. Sci. 33 (11), 535–542. 10.1016/j.kjms.2017.07.001 29050670 PMC11916150

[B221] Zhang X. Y. Sun K. Zhu Q. Song T. Liu Y. (2017a). Ginseng polysaccharide serves as a potential radiosensitizer through inducing apoptosis and autophagy in the treatment of osteosarcoma. Kaohsiung J. Med. Sci. 33 (11), 535–542. 10.1016/j.kjms.2017.07.001 29050670 PMC11916150

[B222] Zhang X. Zhang S. Sun Q. Jiao W. Yan Y. Zhang X. (2018). Compound K induces endoplasmic reticulum stress and apoptosis in human liver cancer cells by regulating STAT3. Molecules 23 (6), E1482. 10.3390/molecules23061482 29921768 PMC6099685

[B223] Zhang Z. Bo J. Xiaoxiang Z. (2004). *In vitro* immune enhancing effect of ginsenoside Rg_3 on peripheral blood lymphocytes in patients treated with tumor radiotherapy. Chin. Pharm. J. 1 (04), 25–28.

[B224] Zhao H. D. Xie H. J. Li J. Ren C. P. Chen Y. X. (2018). Research progress on reversing multidrug resistance in tumors by using Chinese medicine. Chin. J. Integr. Med. 24 (6), 474–480. 10.1007/s11655-018-2910-1 29860581

[B225] Zhao H. Zhang X. Wang M. Lin Y. Zhou S. (2021). Stigmasterol simultaneously induces apoptosis and protective autophagy by inhibiting akt/mTOR pathway in gastric cancer cells. Front. Oncol. 11, 629008. 10.3389/fonc.2021.629008 33708631 PMC7940753

[B226] Zhao J. (2020). Extraction, separation, purification, structural characterization and immune anti-tumor mechanism of ginseng polysaccharide. Changchun: Jilin Agricultural University, 105.

[B227] Zhao X. Y. He Z. Y. Zai S. F. (2020). [Effects of ginsenoside Rg5 on cell cycle and invasion of gastric cancer]. Zhongguo Ying Yong Sheng Li Xue Za Zhi 36 (1), 51–54. 10.12047/j.cjap.5891.2020.011 32476373

[B228] Zhao Y. Chang S. K. Qu G. Li T. Cui H. (2009). Beta-sitosterol inhibits cell growth and induces apoptosis in SGC-7901 human stomach cancer cells. J. Agric. Food Chem. 57 (12), 5211–5218. 10.1021/jf803878n 19456133

[B229] Zheng K. Li Y. Wang S. Wang X. Liao C. Hu X. (2016). Inhibition of autophagosome-lysosome fusion by ginsenoside Ro via the ESR2-NCF1-ROS pathway sensitizes esophageal cancer cells to 5-fluorouracil-induced cell death via the CHEK1-mediated DNA damage checkpoint. Autophagy 12 (9), 1593–1613. 10.1080/15548627.2016.1192751 27310928 PMC5082787

[B230] Zhong J. Chen H. Ye D. Deng Z. Shao J. Han J. (2022). [Molecular mechanism of Ganoderma against gastric cancer based on network pharmacology and experimental test]. China J. Chin. Materia Medica 47 (01), 203–223. 10.19540/j.cnki.cjcmm.20210902.701 35178927

[B231] Zhou Y. Zheng X. Lu J. Chen W. Li X. Zhao L. (2018). Ginsenoside 20(S)-Rg3 inhibits the warburg effect via modulating DNMT3A/MiR-532-3p/HK2 pathway in ovarian cancer cells. Cell. Physiol. biochem. 45 (6), 2548–2559. 10.1159/000488273 29558748

[B232] Zhu H. Liu H. Zhu J. H. Wang S. Y. Zhou S. S. Kong M. (2021a). Efficacy of ginseng and its ingredients as adjuvants to chemotherapy in non-small cell lung cancer. Food Funct. 12 (5), 2225–2241. 10.1039/d0fo03341c 33595586

[B233] Zhu H. Wang S. Y. Zhu J. H. Liu H. Kong M. Mao Q. (2021b). Efficacy and safety of transcatheter arterial chemoembolization combined with ginsenosides in hepatocellular carcinoma treatment. Phytomedicine. 91, 153700. 10.1016/j.phymed.2021.153700 34425474

[B234] Zhu J. Li B. Ji Y. Zhu L. Zhu Y. Zhao H. (2019). β‑elemene inhibits the generation of peritoneum effusion in pancreatic cancer via suppression of the HIF1A‑VEGFA pathway based on network pharmacology. Oncol. Rep. 42 (6), 2561–2571. 10.3892/or.2019.7360 31638231 PMC6826333

[B235] Zhuang J. Yin J. Xu C. Mu Y. Lv S. (2018). 20(S)-Ginsenoside Rh2 induce the apoptosis and autophagy in U937 and K562 cells. Nutrients 10 (3), E328. 10.3390/nu10030328 29518056 PMC5872746

[B236] Zou J. Su H. Zou C. Liang X. Fei Z. (2020). Ginsenoside Rg3 suppresses the growth of gemcitabine-resistant pancreatic cancer cells by upregulating lncRNA-CASC2 and activating PTEN signaling. J. Biochem. Mol. Toxicol. 34 (6), e22480. 10.1002/jbt.22480 32104955

[B237] Zwolak I. (2020). Protective effects of dietary antioxidants against vanadium-induced toxicity: A review. Oxid. Med. Cell. Longev. 2020, 1490316. 10.1155/2020/1490316 31998432 PMC6973198

